# Biofortification of Durum Wheat Grain: Interactions Between Micronutrients as Affected by Potential Biofortification Enhancers and Surfactants

**DOI:** 10.3390/plants14243759

**Published:** 2025-12-10

**Authors:** Despina Dimitriadi, Georgios P. Stylianidis, Ioannis Tsirogiannis, Styliani Ν. Chorianopoulou, Dimitris L. Bouranis

**Affiliations:** 1 Plant Physiology and Morphology Laboratory, Crop Science Department, Agricultural University of Athens, Iera Odos 75, 11855 Athens, Greece; d.dimitriadi@aua.gr (D.D.); g.stylianidis@aua.gr (G.P.S.); tsiros02@yahoo.com (I.T.); s.chorianopoulou@aua.gr (S.Ν.C.); 2 Karvelas AVEE, 80th km Athinon-Lamias, 32200 Ypato, Greece; 3 PlanTerra, Institute for Plant Nutrition and Soil Quality, Agricultural University of Athens, Iera Odos 75, 11855 Athens, Greece

**Keywords:** sulfur-assisted agronomic biofortification, specialty fertilizers, micronutrients metallome, zinc, iron, manganese, copper, sulfate, additives, cysteine, methionine, alcohol ethoxylate, organosilicon ethoxylate

## Abstract

Wheat possesses inherently low concentrations and bioavailability of the essential micronutrients (EMis) zinc (Zn), iron (Fe), manganese (Mn), and copper (Cu), limiting its capacity to sufficiently address human nutritional requirements. Biofortification of wheat with EMis through agricultural methods is a strategy aimed at addressing EMi deficiencies in human populations that emphasize cost-effectiveness and sustainability. All EMis are usually applied foliarly as sulfates, which indicates sulfur (S)-assisted biofortification. The formation of EMi complexes provides solubility as well as protection during long-distance transport. Several small molecules are possible candidates as ligands—the S-containing amino acids cysteine and methionine among them—linking EMi homeostasis to S homeostasis, which represents another aspect of S-assisted biofortification. In this study, we delve into the S-assisted agronomic biofortification strategy by applying sulfate micronutrients coupled with a sulfur-containing amino acid and we explore the effect of the selected accompanying cation (Zn, Fe, Mn, or Cu) on the EMi metallome of the grain, along with the biofortification effectiveness, whilst the type of the incorporated surface active agent seems to affect this approach. A field experiment was conducted for two years with durum wheat cultivation subjected to various interventions at the initiation of the dough stage, aiming to biofortify the grain with EMis provided as sulfate salts coupled with cysteine or methionine as potential biofortification enhancers. The mixtures were applied alone or in combination with commercial surfactants of the organosilicon ethoxylate (SiE) type or the alcohol ethoxylate (AE) type. The performance of two relevant preparations, FytoAmino-Bo (FABo) and Phillon, has been studied, too. The interventions affected the accumulation of the EMi metallome into the grains, along with the interactions of the EMis within this metallome. Several interventions increased the EMi metallome of the grain and affected the contribution of each EMi to this metallome. Many interventions have increased Zn and Fe, while they have decreased Mn and Cu. An increase in Zn corresponded (i) to a decrease in Cu, (ii) to an increase or no increase in Fe, and (iii) to a variable change in Mn. Cys increased the metallome by 34% and Zn and Fe within it. ZnSO_4_ and FeSO_4_ increased the metallome by 5% and 9%, whilst MnSO_4_ and CuSO_4_ increased the metallome by 36% and 33%, respectively. The additives improved the contribution to increasing the metallome in most cases. Without surfactant, the efficacy ranking proved to be MnSO_4_ > CuSO_4_ > ZnSO_4_ > FeSO_4_. The use of SW7 sustained the order CuSO_4_ > MnSO_4_ > ZnSO_4_ > FeSO_4_. The use of Saldo switched the order to CuSO_4_ > ZnSO_4_ > FeSO_4_ > MnSO_4_. In the case of Phillon, the order was CuSO_4_ > FeSO_4_ > ZnSO_4_ > MnSO_4_. The effect of Cys or Met was case-specific. The differentiations in the intensity of both the agronomic performance (grain weight, grain weight per spike, and yield) and the biofortification performance (concentrations vs. accumulations of each EMi within the grain) among the various combinations of EMis and additives are depicted by adopting a grading scale, which highlighted the intensity of the acclimation reaction of the biofortified grain to the applied intervention.

## 1. Introduction

Wheat possesses inherently low concentrations and bioavailability of the essential micronutrients (EMis) zinc (Zn), iron (Fe), manganese (Mn), and copper (Cu), limiting its capacity to sufficiently address human nutritional requirements. Among this EMi metallome, Fe and Zn are most commonly deficient in the diets of populations in developing countries. These minerals play vital roles in various human metabolic processes. Although Cu and Mn deficiencies are not prevalent worldwide, these EMi are nonetheless critical for human growth and development [1]. Moreover, Zn, Fe, Mn, Cu, along with B, and Mo are key EMis for higher plants, as they play vital roles in promoting plant growth due to their essential functions. EMi deficiencies tend to occur in areas where soils have low concentrations of plant-available EMis. Since agriculture-based foods are a primary source of human nutrition, the link between soil nutrients, crops, and human health is clear [2,3,4]. Food systems are increasingly globalized and interdependent [5]. The International Fertilizer Industry Association (IFA) defines “nutrition-sensitive agriculture” as an approach to agricultural development that emphasizes nutritionally rich foods, dietary diversity, and food fortification to address malnutrition and micronutrient deficiencies. Improving the nutrition sensitivity of agriculture involves adjustments in approaches to thinking, planning, implementation, and collaboration [6].

Biofortification of staple cereals with EMis through agricultural methods is a strategy aimed at addressing EMi deficiencies in human populations which emphasizes cost-effectiveness and sustainability. Biofortification programs primarily focus on developing high-yield wheat genotypes with higher EMi concentrations in their grains. Biofortification serves as a targeted intervention strategy to address EMi deficiencies through the enhancement of the nutritional content in crop plants by employing two key approaches: (i) genetic biofortification, which utilizes conventional plant breeding or genetic engineering techniques to enhance nutrient concentrations; (ii) agronomic biofortification, which involves optimizing fertilizer application methods [1]. Plant breeding and fertilizer management approaches are both complementary and synergistic in advancing agricultural productivity. The integration of these agricultural methods has yielded both additive and synergistic effects on grain EMi concentrations [2,7,8].

Micronutrient deficiencies typically arise in regions where soils contain inadequate concentrations of plant-available micronutrients [9]. Deficiencies of Zn and Fe are widespread, with these EMi deficiencies affecting over one-third of the global population. Agricultural systems have typically not been designed to improve human nutrition and health. The principal aim has traditionally been to optimize food production to mitigate the risks of hunger and famine. Incorporating EMi-enriched fertilizers represents an effective strategy to address hunger and malnutrition.

In the literature, all EMis are usually applied foliarly as sulfates. Among the different forms of Zn fertilizers that were tested, the application of Zn as ZnSO_4_ was the most effective in increasing grain Zn content, compared to other forms of Zn [10]. Zn, Fe, Mn, and Cu are EMis that are physiologically useful for plants, animals, and humans. Both their deficiency and excess cause various malfunctions in each organism. These elements comprise a metallome of central contribution to plant functioning. For humans to consume food of quality, the soil, plant, and human chain requires very efficient management of the EMi metallome. Within plants, EMi management includes acquisition, transport, translocation, utilization, and re-translocation. Synchronization of co-functioning of the biological systems that will handle the EMi is required. Understanding of the EMi metallome homeostasis requires detailed knowledge of the dynamics of this network in plants. The fact that EMi are provided as sulfates indicates sulfur-assisted biofortification and the interaction of this metallome with sulfur (S) has been discussed [11].

The formation of EMi complexes provides solubility as well as protection during long-distance transport, as the EMi atom is surrounded by ligands. These chemical species donate several electron pairs to the EMi to form the complexes. Possible candidates as ligands are several small molecules, the S-containing amino acids Cys, and Met among them. Nicotianamine (NA) has been shown to participate in the transport of the EMi metallome, i.e., Fe, Cu, Mn, and Zn [12]. NA is produced by methionine and acts as a chelator through its carboxyl groups, possessing a special role in the interaction between S and EMi homeostasis. NA is synthesized by NA synthase (NAS) from S-adenosyl-L-methionine. It is a ubiquitous metal chelator in all plants. NA is a Fe chelator, and it has demonstrated its ability to bind both Fe and Cu. It is believed to play a primary role in EMi homeostasis, and therefore it links EMi homeostasis to S homeostasis [13]. This information represents another aspect of S-assisted biofortification.

In foliar applications, a wetter or surfactant (surface active agent) is usually incorporated. Studies showed that ZnSO_4_ could be mixed with some wheat herbicides, insecticides, and fungicides without affecting the effectiveness of foliar application for increasing grain Zn concentration. This would increase the possibility that farmers may be willing to apply ZnSO_4_ in their fields by reducing the cost and time of application. To our knowledge, there is no literature deepening on the use of surfactants in applying biofortification solutions. Surfactants are incorporated into agrochemical formulations to enhance the biological efficiency of foliar sprays by improving the wetting behavior of the spray and/or the penetration of the active ingredients into the leaf tissues. Penetration-accelerating surfactants are known to increase the cuticular permeability and may submit the cuticular barrier to water loss [14,15,16]. Among the various types of surfactants, two are in common use in the experimental area: alcohol ethoxylate (AE) surfactants or organosilicon-based ethoxylate (SiE) ones. Ethoxylated surfactants may improve spray retention and leaf wetting, whilst they may also increase cuticular permeability [17]. Generally, surfactant effects are species- and compound-specific [18].

Timing of foliar EMi application is an important factor determining its effectiveness in increasing grain EMi concentration; grain EMi increases are most likely when foliar Zn fertilizers are applied to plants at a late growth stage. Ozturk et al. (2006) [14] studied changes in grain Zn concentration in wheat during the reproductive stage and found that the highest concentration of grain Zn occurs during the milk stage of grain development. Foliar application of Zn during reproductive growth seems to be more effective in increasing grain Zn concentration than spraying of Zn at earlier growth stage. A working question that arises is whether we receive the same result or not, in case we apply one of the other three EMi at this stage.

In this study we delve into the S-assisted agronomic biofortification strategy by applying a sulfate micronutrient coupled with a sulfur-containing amino acid, and we deepen into the effect of the selected accompanying cation (Zn, Fe, Mn, or Cu) on this EMi metallome, along with the biofortification effectiveness. Does the type of the incorporated surface active agent affect this approach? A field experiment was conducted for two years with durum wheat cultivation subjected to various interventions at the initiation of dough stage, towards biofortifying the grain with EMi. EMi were provided as sulfate salts (EMiSO_4_), coupled with cysteine (Cys) or methionine (Met) as potential biofortification enhancers. The mixtures were applied alone or in combination with the commercial surfactants SW7 (of SiE type) and Saldo (of AE type). Moreover, we have also studied the performance of two preparations: FytoAmino-Bo (FABo) and Phillon. FABo contains ZnSO_4_, combined with arginine, ethanolamine borate and molybdate as MoO_4_. Arginine is split into urea and ornithine by arginase, and then urea produces ammonium by urease. Hence, apart from Zn, ammonium is also provided at the action place, along with boron and molybdenum. Moreover, ethanolamine, along with ornithine, both contribute to flag leaf and spike strengthening. Phillon has been designed as specialty surfactant that combines a wetting agent being a surfactant of AE type and emulsifier, coupled with soybean oil, in an attempt to further support the action of Cys or Met applied as emulsions.

## 2. Results

### 2.1. S-assisted Biofortification of Durum Wheat Grain with Zinc

The reference range for grain Zn concentration was 26.42–27.98 mg kg^−1^ (Table 1 and Table S1), for grain Fe concentration was 23.1–24.6 mg kg^−1^, for grain Mn concentration was 9.2–9.6 mg kg^−1^, and for grain Cu concentration was 4.8–6.6 mg kg^−1^. Toward visualization of the intensity of the changes caused by the interventions, a grading scale has been adopted. The calculated percentage differences of the means compared to the corresponding control (Δ%) were categorized into four positive and four negative groups: A+ (0 to 19%), B+ (20 to 39%), C+ (40 to 59%), and D+ (>60%), A− (−1 to −19%), B− (−20 to −39%), C− (−40 to −59%), and D− (<−60%). The interventions with Zn presented the following effectiveness (Table 1 and Table S1). The intervention with ZnSO_4_ increased Zn concentration by 35%. In fact, all combinations of ZnSO_4_ presented increases (B+, C+).The combination of ZnSO_4_ with Cys or Met increased Zn concentration by 50% and 41%, respectively. The use of SW7 reduced the aforementioned increases (by 2%, 8%, and 14%, respectively). The use of Saldo increased further the increase in Zn concentration in combination with ZnSO_4_, whilst this was not true for the combinations ZnSO_4_/Cys or ZnSO_4_/Met. The combination ZnSO_4_/Cys/Phillon provided the highest increase in Zn concentration by 58%.

The intervention with ZnSO_4_ did not affect grain Fe concentration. Instead, combinations of ZnSO_4_/Cys and ZnSO_4_/Met increased grain Fe concentration by 26% and 18%, respectively. The intervention with ZnSO_4_ decreased grain Mn concentration and Cu concentration by −22% and −30%, respectively. The combination of ZnSO_4_/Cys increased Mn concentration by 28%. No other increases were found with regard to Mn concentration and Cu concentration.

### 2.2. S-assisted Biofortification of Durum Wheat Grain with Iron

The intervention with FeSO_4_ did not affect Fe concentration or Mn concentration (Table 2 and Table S2). In contrast, it increased Zn concentration by 26% and decreased Cu concentration by −32%. The combination with Cys did not affect Fe concentration or Mn concentration. In contrast, it increased Zn concentration by 40% and decreased Cu concentration by −32%. The combination with Met increased Fe concentration by 14% and Zn concentration by 37%, whilst decreasing Mn concentration by −30% and Cu concentration by −28%. The combination of FeSO_4_ with Saldo increased Fe concentration by 12%, Zn concentration by 25%, and Mn concentration by16%, whilst decreasing Cu concentration by −28%. The same result was provided by the combination of FeSO_4_ with Phillon; it increased Fe concentration by 23%, Zn concentration by 44%, and Mn concentration by 16%, whilst decreasing Cu concentration by −27%. The combination of FeSO_4_/Cys with SW7 increased Fe concentration by 4%, Zn concentration by 17%, and Mn concentration by 15%, whilst decreasing Cu concentration by −41%.

### 2.3. S-assisted Biofortification of Durum Wheat Grain with Manganese

The intervention with MnSO_4_ increased Mn concentration by 22%, Zn concentration by 53%, and Fe concentration by 28%, whilst it decreased Cu concentration by −36% (Table 3 and Table S3). The combination of MnSO_4_ with Cys decreased Mn concentration by −8%, increased Zn concentration by 10%, did not affect Fe concentration, and decreased Cu concentration by −39%. The combination of MnSO_4_ with Met decreased Mn concentration by −6%, did not affect Zn concentration and Fe concentration and decreased Cu concentration by −41%. The combination of MnSO_4_ with surfactants increased Mn concentration, Zn concentration, and Fe concentration and decreased Cu concentration. The highest concentrations were achieved by MnSO_4_/SW7, increasing Mn concentration by 53%, Zn concentration by 71%, and Fe concentration by 38%, whilst Cu concentration was decreased by −30%.

### 2.4. S-assisted Biofortification of Durum Wheat Grain with Copper

The intervention with CuSO_4_ decreased Cu concentration by −33% and Mn concentration by −30%, and increased Zn concentration by 38% and Fe concentration by 50% (Table 4 and Table S4). All combinations increased Zn concentration and Fe concentration and decreased Mn concentration and Cu concentration. The combination of CuSO_4_ with Cys increased Zn concentration by 30% and Fe concentration by 19%, and decreased Mn concentration by −18% and Cu concentration by −28%. The combination of CuSO_4_ with Met increased Zn concentration by 59% and Fe concentration by 20%, while it decreased Mn concentration by −10% and Cu concentration by −33%.

### 2.5. S-assisted Biofortification of Durum Wheat Grain with FABo

The intervention with FABo increased Zn concentration by 73%, Fe concentration by 32%, and Mn concentration by 20% and decreased Cu concentration by −36% (Table 5 and Table S5a). Combinations of FABo with surfactants decreased the result achieved by FABo alone. The combination of FABo with Cys increased Zn concentration by 13% and Fe concentration by 21%, and decreased Mn concentration by −19% and Cu concentration by −66%. The combination of FABo with Met increased Zn concentration by 37% and Fe concentration by 27%, and decreased Mn concentration by −19% and Cu concentration by −47%. The combination of FABo/Met/Saldo increased Zn concentration by 44%, Fe concentration by 30%, and Mn concentration by 9% and decreased Cu concentration by −56%. The first experimental year, FABo- was tested, too, i.e., FABo without arginine, in order to study the effect of arginine on the results (Table S5b). The results show that FABo- presented behavior close to that of ZnSO_4_.

### 2.6. The Contribution of the S-Amino Acids and Surfactants on the Biofortification Process: A Role as Intervention Assistants?

The effects of interventions containing additives without EMis are presented in (Table 6 and Table S6), namely the surfactants alone, Cys or Met alone, and their combinations with surfactants. Surfactants alone did not affect the concentrations of the EMis. Intervention with Cys alone resulted in an increase in grain Zn concentration by 56% and Fe concentration by 25%, and simultaneously in a decrease in grain Mn concentration by −49% and Cu concentration by 26%, respectively. A similar effect was observed for the intervention with Met alone: changes in Zn concentration by 12%, Fe concentration by 15%, Mn concentration by −18%, and Cu concentration by −33%. The results received by their combinations with surfactants were case-specific. In general, these combinations increased Zn concentration and in most cases Fe concentration slightly, whilst decreasing Mn concentration and Cu concentration.

### 2.7. EMi Accumulation per Grain and Their Contribution to EMi Metallome

Concentration is a ratio (amount divided by biomass); hence, grain weight and yield data is needed to distinguish whether the determined concentrations and their changes are a result of enhanced nutrient accumulation (true biofortification) or reduced grain weight (concentration artifact). These data are presented in Table 7a,b, Table 8 and Tables S7 and S8. The grain weight remained statistically unchanged; however, in most cases we see tendencies towards increasing.

As regards the grain weight per spike, we see that decreases or tendencies to decrease are observed. This is reflected in the yields of the corresponding plots per treatment (Table 8 and Table S8).

The accumulated amounts of each EMi, expressed in μmol of EMi per grain, are presented in Table 9a,b and Table S9. The control was characterized by an equal μmol of Zn and Fe accumulated; Zn:Fe:Mn:Cu = 0.023:0.023:0.009:0.005; the sum of EMi was 0.061 μmol; and the percentage contribution of each EMi to the metallome was Zn:Fe:Mn:Cu = 38%:39%:15%:8%. The EMi accumulation per grain was affected by the interventions. The molar contribution of Cu was reduced. Exceptions were found when CuSO_4_ was applied alone or with additives. The case of CuSO_4_/Cys/Phillon increased all ΕΜis. The sum was 0.107 μmol per grain and the percentage increase for Zn:Fe:Mn:Cu was 367%:409%:134%:112%.

**Figure 1:**
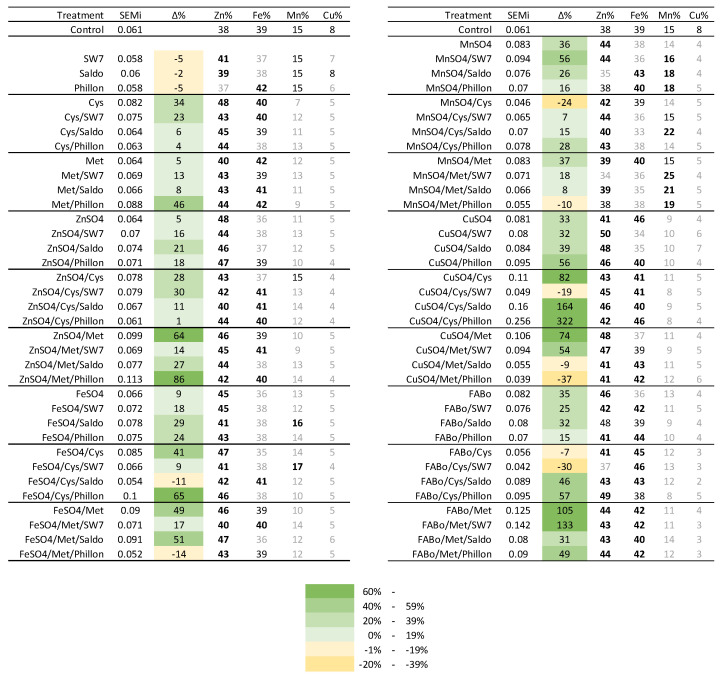
The sum of the EMi metallome (SEMi; in μmol) per grain is given, along with the percentage contribution of each EMi to this metallome (experimental year 2022–2023). Δ%: relative percentage difference between the treatment and the control. Visualizing the effects, the grading scale is depicted in color, whilst in bold the increases and in gray the decreases in percentage contribution are depicted.

Several interventions increased the EMi metallome of the grain and affected the contribution of each EMi to this metallome. This response is visualized in Figure 1, where it is apparent that many interventions increased Zn and Fe, while they decreased Mn and Cu. Cys increased the metallome by 34% and Zn and Fe within it. ZnSO_4_ and FeSO_4_ increased the metallome by 5% and 9%, whilst MnSO_4_ and CuSO_4_ increased the metallome by 36% and 33%, respectively. The additives improved the contribution to increasing the metallome in most cases; hence, the cases that presented decreases in the metallome were FeSO_4_/Cys/Phillon, FeSO_4_/Met/Phillon, MnSO_4_/Cys, MhSO_4_/Met/Phillon, CuSO_4_/Cys/SW7, CuSO_4_/Met/Saldo, CuSO_4_/Met/Phillon, FABo/Cys, and FABo/Cys/SW7. Instead, the highest increases include Met/Phillon, ZnSO_4_/Met, ZnSO_4_/Met/Phillon, FeSO_4_/Cys/Phillon, CuSO_4_/Cys, CuSO_4_/Cys/Saldo, CuSO_4_/Cys/Phillon, CuSO_4_/Met, and FABo/Met/SW7.

## 3. Discussion

Agronomic biofortification involves foliar applications of EMis, and the interventions are S-assisted in two ways: EMis have been used as sulfates and the S-containing amino acids Cys or Met have been used as additives. A durum wheat crop was established in soil that was marginally low in all four EMi contents, and in several cases, we managed to increase the grain Zn concentration simultaneously with the Fe concentration and Mn concentration; however, it was not possible to increase the Cu concentration. Moreover, the achieved increases in the grain Zn, Fe, and Mn concentrations were achieved by various EMiSO_4_ formulations. With a view to preparing effective specialty EMi fertilizers, the contribution of the S-containing amino acids Cys and Met was studied based on the fact that Cys or Met may act as EMi chelators or/and NA precursors.

**Figure 2:**
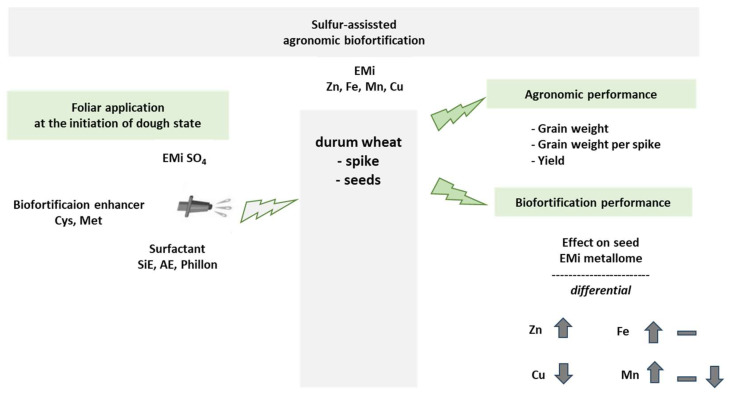
Summary of the experimental approach and its effects on the agronomic and biofortification performances. The effects of the interventions on these performances were differential, and the intensities of the interactions are summarized in Table 10. EMi: essential micronutrients; Cys: cysteine; Met: methionine; SiE; organosilicon ethoxylate surfactant; AE: alcohol ethoxylate surfactant.

Towards highlighting the range of the response intensity, a synopsis of the effect of the interventions on the grain metallome is summarized in Figure 2 and presented in Table 10. The adopted grading scale assisted in providing useful comparisons and from the used interventions we have learned the following: Without surfactant, the ranking proved to be FABo > MnSO_4_ > CuSO_4_ > ZnSO_4_ > FeSO_4_. Especially, FABo (group D+ for Zn concentration) and MnSO_4_ (C+ for Zn concentration) increased all three concentrations: Zn, Fe, and Mn. CuSO_4_ (B+) increased Zn concentration and Fe concentration, whilst ZnSO_4_ (B+) and FeSO_4_ (B+) only Zn concentration. Cys proved to be a strong enhancer for Zn concentration and Fe concentration, but a strong reducer for Mn concentration and Cu concentration. Met proved to be a medium enhancer for Zn concentration and Fe concentration, a light reducer for Mn concentration, and a strong reducer for Cu concentration.

Moreover, in an attempt to select treatments that balance biofortification and agronomic performance, the following steps were taken: (i) the interventions that presented a positive agronomic performance with a positive grading scale in both grain weight and grain weight per spike were marked, and (ii) it was checked whether the yield GS was positive, too. The cases ZnSO_4_/Saldo, ZnSO_4_/Cys/SW7, ZnSO_4_/Cys/Saldo, ZnSO_4_/Met/SW7, MnSO_4_, MnSO_4_/Saldo, MnSO_4_/Cys, and MnSO_4_/Met/Phillon were promoted. Then, the EMi accumulations were marked and evaluated, along with the SEMi. However, certain cases presented negative accumulations in all EMis (MnSO_4_?Cys and CuSO_4_/Met/Saldo), as well as positive accumulations in all EMis (CuSO_4_/Cys, CuSO_4_?Cys/Saldo, and CuSO_4_/Cys/Phillon).

Most published studies on the agronomic biofortification of food crops using foliar spraying have concentrated on applying one EMi, or occasionally two [2]. EMis are essential for increasing wheat yields, and crop production has been increased significantly with the use of EMis [19]. Τo increase the grain Zn concentration, ZnSO_4_ is usually applied to the crop, whilst for increasing grain Fe concentration, FeSO_4_ is the choice. Across most wheat-growing areas, grain Zn concentrations typically fall within the range of 20 to 30 mg kg^−1^ and it is recommended that grain Zn content be maintained between approximately 40 and 50 mg kg^−1^ [2]. Fertigation with Zn salts like ZnSO_4_ typically increases grain Zn [20]. Applying Fe and Zn to leaves increases their levels in wheat [21]. The foliar application of Fe fertilizers was determined to be ineffective in increasing wheat grain Fe concentration to the desired target level [22,23].

The grain development stage is critical for the effectiveness of foliar application [14,24]. Velu et al. (2014) [10] showed changes in the Zn concentration of the endosperm part of wheat seeds from plants sprayed with ZnSO_4_ at different growth stages in the field. Late-season (i.e., at the milk and dough stage) foliar application of Zn increased the concentration in the starchy endosperm by up to 3-fold. Several studies have examined various Zn application methods, timing, molecular forms, and genes involved in Zn transport and accumulation [25,26]. In wheat crops, there are three main critical periods when the greatest need for nutrients is observed. (1) Budding: Leaf feeding stimulates the growth of the main shoot, the establishment of side shoot buds in the axils of the germinating leaves, and the growth of the germ system. (2) Tillering: The emergence of tillers activates morphophysiological processes, ensuring the growth of a secondary root system. (3) The flag leaf stage, marking the beginning of the emergence of the spikes: Leaf treatment, in this stage, qualitatively improves the processes of flowering, grain formation, and development [27]. Despite these potential benefits, the application of EMi fertilizers in wheat fertilization and stress management may be accompanied by risks to the environment, non-target plants, beneficial soil microbes, and other life forms that may be affected if the fertilizers are not used judiciously [27]. It has been reported [28] that root growth in wheat was improved by spraying EMis which led to increase in the uptake of macro- and micronutrients. Moreover, there is an increase in protein percentage of seed and yield components due to foliar application [19].

Since foliar application was performed on the upper part of the aerial part that includes the spike and the flag leaf, remobilizations of the nutrients from the flag leaf to the grain are expected to take place. The metals when not in equivalent ratios show the potential risk of interactions affecting absorption and bioavailability, translocation within plants, storage, and related physiological effects. Plants need to supply appropriate amounts of each of these EMi to the precise target apo-metalloproteins and meanwhile avoid adventitious metal binding to non-target metal binding sites or other cellular compounds [29]. Cu’s fluxes and interactions with other EMi (Fe, Mn, and Zn) affect the growth and yield of wheat plants, while Cu excess may induce the deficiency of other micronutrients and adversely affect the yield [30,31]. Despite the significant literature on individual metal homeostasis on these EMis, gaps remain in our understanding of how they overlap in plants and the related physiological effects. More molecular players are still needed to be identified in this metal crosstalk. Deeper understanding of the complexities with which these EMis interact and influence each other would improve the biofortification strategy for grains enriched with EMis.

There is available literature discussing the interactions between EMis in wheat. An antagonism exists between Fe and Mn, as well as between Zn and Cu [29]. Rai et al. (2021) [29] have discussed the role of EMis in plant and expanded on Fe homeostasis and its crosstalk with Cu, Zn, and Mn. Understanding the complexities of the interaction between Fe and other EMis and how it defines the health of plants facilitates improved plant growth strategies in soils with low/high levels of these metals, with implications for agriculture and phytoremediation. Maintaining an optimum level of these EMis in the plant requires balanced activities of transporters that mediate import into the cell, proper distribution to where it is needed and stored, and use in metalloproteins and metalloenzymes within the cell [29,32,33,34,35,36,37].

The transportation of the EMis existed before application within the plant body was assisted or provided by the applications. In any case, the redistribution of EMis took place through movement in the phloem. Traveling towards the seed, Zn and Fe transport in the phloem, mainly chelated with NA [38,39]. The accumulation of NA in plant organs changes under metal deficiency or excess [12]. Although NA biosynthesis can be induced in vivo by various metals, it is mainly involved in the detoxification and transport of Fe, Zn, Ni, Cu, and Mn. Although there are no details available for Mn allocation mechanisms to the seed, it is known that Mn is mainly transported inside the plant body as Mn^2+^ through various transporters. On the other hand, Mn has low phloem mobility, whilst chelation with NA is considered feasible but chelation is not the main route for Mn distribution [40]. The work of Zou et al. (2025) [41] regarding the genetic basis for Cu accumulation in wheat grains indicated that candidate proteins for Cu entering the wheat grain are transporters mainly involved in the transport of divalent Cu, and not the chelated form. Considering the above, it seems reasonable that any application that provides the flag leaf and spike with a precursor molecule of NA (in the form of either inorganic or organic S) will favor Fe and Zn translocation and distribution to the grain. Subsequently, possibly due to competition phenomena, Mn and Cu are “sidelined” EMis, which may explain our results.

The findings highlight the fact that the increase in Zn concentration is accompanied by reverberations in the concentrations of all three other EMis. The molar ratio achieved by each intervention showed that the metallome is affected by the treatment and the result is ingredient-specific. The tendencies revealed only increases in Zn concentration, increases or no increases in Fe concentration, increases or decreases in Mn concentration, and only decreases in Cu concentration. It is clear that an increase in Zn corresponds (i) to a decrease in Cu, (ii) to an increase or no increase in Fe, and (iii) to a variable change in Mn. The use of SW7 promoted the order CuSO_4_ > MnSO_4_ > ZnSO_4_ > FeSO_4_. The use of Saldo switched the order to CuSO_4_ > ZnSO_4_ > FeSO_4_ > MnSO_4_. The use of Phillon promoted FeSO_4_ and the order was CuSO_4_ > FeSO_4_ > ZnSO_4_ > MnSO_4_. The effect of Cys or Met was case-specific. The effectiveness of nutrient absorption by plants is influenced by factors including leaf anatomy, the timing of application, soil pH, and prevailing climatic conditions [42,43]. The beneficial impact of soil or foliar Fe fertilization on grain Fe concentration is observable predominantly when plants possess adequate nitrogen (N) nutrition [44,45,46]. Enhanced N nutrition facilitates root uptake, shoot transport, and seed deposition of both Zn and Fe. That was the rationale behind the use of arginine. Foliar application of FABo provided simultaneously Zn, B, Mo, and NH_4_^+^; hence, it seems to be suitable as a specialty EMi fertilizer proper for nutrition-sensitive agriculture.

Discussing the effect of FABo and Phillon on the efficacy of Cys and Met as biofortification enhancers (or reducers in the case of Cu concentration), the following information has been gained. The product FABo was first in the ranking without the use of a surfactant (group D+ for Zn concentration, where it also increased simultaneously Fe concentration (B+) and {Mn] (B+) but not Cu concentration (B−). Its combination with Met or Cys reduced it in group B+ and A+ for Zn concentration, respectively. FABo/Cys reduced Cu concentration by 66% (D−). The use of SW7 switched the order: FABo/Met/SW7 was found in C+, FABo/SW7 in B+, and FABo/Cys/SW7 in A+ group for Zn concentration. The use of Saldo also reversed the ranking: FABo/Saldo and FABo/Met/Saldo were in the C+ group, and FABo/Cys/Saldo in the B+ group where Cu concentration was reduced by 72% (D−). As regards Phillon, an AE surfactant that can act as an emulsifier too was incorporated into the product oil of plant origin in order to potentially support the action of Cys or Met. When Phillon was used, FABo/Cys/Phillon was upgraded in group D+ for Zn concentration, FABo/Met/Phillon in C+ (with Cu concentration reduced in D−), and FABo/Phillon in B+. In all cases, Fe concentration was found in the B+ group. This picture suggests that the lipophilicity of the two S-containing amino acids [47] seems to have been influenced by the soybean oil content of Phillon, a characteristic that seems to contribute to the efficacy of the interventions and merits further research. It is also worth noting that the strongest decrease in Cu concentration was observed for FABo/Cys (−66%), FABo/Cys/Saldo (−72%), and FABo/Met/Phillon (−61%), an observation that merits further research. All three cases increased Zn concentration and Fe concentration and decreased Mn concentration (all in group A− for Mn concentration).

When EMi fertilizers are combined with phytoprotectants, it is highly possible that surfactants will be added to the application solution. To our knowledge, there is no relevant literature studying such approaches for grain biofortification reasons. Alcohol ethoxylates are used as surfactants in a wide variety of agrochemical formulations to enhance the effectiveness of the active constituents. Alcohol ethoxylates belong to the class of compounds which are synthesized via the reaction of a fatty alcohol and ethylene oxide, resulting in a molecule that consists of two parts: the first part is carbon-rich, fatty alcohol and the second part a hydrophilic polyoxyethylene chain [48]. Saldo contains isodecyl alcohol ethoxylate. As regards SiE, the silicon–oxygen bonds are hydrophobic, whilst the ethoxylated clusters are hydrophilic, creating a wetting agent that spreads quickly, thus covering a large surface area, greater than conventional surfactants. Silicone surfactants undergo a relatively rapid hydrolytic cleavage in the environment, as do linear silicone polymers, to give monomers that are more slowly converted by oxidation back to water, CO_2_, and sand [49,50]. Generally, surfactant effects are species- and compound-specific [48]. Coupling Cys or Met with SiE or AE has been used in the biofortification of broccoli [51].

The use of SiE increased the use of EMi by increasing Zn concentration. CuSO_4_/SW7 provided the highest Zn concentration (D+), whilst in decreasing order MnSO_4_/SW7 (D+), ZnSO_4_/SW7 (B+), and FeSO_4_/SW7 (B+) also provided a strongly increased Zn concentration. MnSO_4_/SW7 is recommended, as it increases Zn concentration, Fe concentration, and Mn concentration simultaneously. MnSO_4_/SW7 increased Mn concentration by 53%. The combination with Cys was not strong for FeSO_4_/SW7 (A+) and FABo/SW7 (A+). Cys/SW7 (B+) reduced the efficacy of the intervention with Cys alone. Met in combination with CuSO_4_/SW7 (C+), FABo/SW7 (C+), and ZnSO_4_/SW7 (B+) provided good results. It is worth mentioning that Fe concentration was significantly increased for MnSO_4_/SW7 (38%), ZnSO_4_/Cys/SW7 (37%), FABo/Met/SW7 (35%), FABo/Cys/SW7 (30%), and FABo/SW7 (27%).

The use of AE ranked CuSO_4_/Saldo (group D+ for Zn concentration), FABo/Saldo (C+), ZnSO_4_/Saldo (C+), and FeSO_4_/Saldo (B+) as good combinations. Especially, CuSO_4_/Saldo was the only intervention that increased Cu concentration (by 10%), along with Zn concentration and Fe concentration but not Mn concentration. Fe concentration was increased by several interventions. FABo/Met/Saldo, ZnSO_4_/Cys/Saldo, FeSO_4_/Saldo, and MnSO_4_/Saldo increased all three EMis of Zn concentration, Fe concentration, and Mn concentration.

We have shown [52] that the spray application of the treatments affected the spike’s developmental program, which switched to the acclimation process. The effect of the interventions on the developmental acclimation program of the treated spike has been presented and discussed. The action of this program provided grains of similar weight, regardless of the intervention. The described alterations suggest that signaling is affected by the treatments. The alterations observed in spike architecture may stem from disruptions in hormonal equilibrium induced by foliar sprays. Foliar EMi application can influence hormonal balance by modifying the activity of enzymes involved in hormone biosynthesis: for instance, Zn-dependent auxin synthesis and Fe-dependent synthesis of gibberellins and cytokinins. Iron and Cu are redox-active metals that can switch between two oxidation states and are known to have a pro-oxidant role and be directly involved in ROS generation. On the contrary, Zn and Mn mostly have roles in ROS scavenging processes. Moreover, the fact that in various treatments with Fe and Cu, Cys is also present in the formulation points out its role as a superior ligand for both metals. Incorporating the findings of this work into the presented acclimation program, it seems that the acclimation program affects the remobilization of stored minerals in vegetative tissues during grain filling, whilst the result is defined by the dilution effect due to the biomass allocated in the spike chaff. In this acclimation response, the sum of the accumulated EMi has been increased in several cases with alteration of the contribution of each EMi to the metallome.

Given the fact that in most cases yield decreased due to the interventions, S-assisted fertilization could be used to improve this acclimation response. The fertilization used in the experiment was the conventional one used by the farmers of the area. We have shown [53] that the use of urea plus ammonium sulfate, coated by elemental sulfur plus the urease inhibitor NBPT by means of molasses and glycerol as coating agents provides significant increases in the yield of wheat. The effect of elemental sulfur as a fertilizer ingredient on the mobilization of Fe from the Fe pools of a calcareous soil cultivated with durum wheat and the crop’s iron and sulfur nutrition [54], along with the impact of elemental sulfur on the rhizospheric bacteria of such crops [55], has been studied. Hence, the combination of S-assisted fertilization and biofortification seems to be an improved strategy that could be the aim of further research.

## 4. Materials and Methods

Two field experiments were carried out in 2021–2022 and 2022–2023, in the area of the Experimental Fields of the Agricultural University of Athens, at the location of Aliartos, Viotia, Greece (Coordinates: 38°23′48.3″ N, 23°05′23.8″ E). The soil was found to present the following characteristics: clay 42%, loam 30.3, sand 27.2%, calcium carbonate 50.4%, organic matter 2.8%, pH 8.4, K 110 ppm, P 9.2 ppm, exchangeable Mg 528 ppm, Fe 5.1 ppm, Mn 3.7 ppm, Zn 0.72 ppm, Cu 0.32 ppm, and B 0.4 ppm.

The durum wheat variety studied was Don Matteo. The agronomic program included the following: basic fertilization, NOVATEC 20 − 20 − 5 + B + Zn (Compo Expert Hellas, Athens, Greece); herbicide application, Corello 75 wg (Corteva Agriscience, Athens, Greece) and Mustang (DOW Agriscience, Athens, Greece); fungicide application, Madison (Bayer Cropscience, Athens, Greece); top dressing fertilization, OMEGA 26 − 0 − 0 (Hellagrolip, Kavala, Greece); no irrigation was applied to the crop. The dates of the experiment’s agronomic works are provided in Table 11. The experimental plots were formed with dimensions of 3 m × 1.5 m and 1 m distance between them. The interventions followed with foliar application at the developmental stage Z80, using a randomized block design. The volume of spray liquid was 1 L per experimental plot, while the sprays took place during the afternoon–early evening hours (18:00–21:30). Per square meter, 72–77 plants were hosted carrying 2.4–2.7 spikes and 4–6 tillers per plant. During harvest, 10 heads were sampled from the main stems per experimental plot. Per treatment, three plots were studied, and in total 30 spikes were analyzed. Three biological samples were prepared by mixing equal amounts of grains from each of the three plots and two chemical replications per biological sample were performed. Samples were oven-dried (80 °C) prior to chemical analysis. Zn, Fe, Mn, and Cu were determined after digestion of the tissues in a mixture of sulfuric acid and hydrogen peroxide [56]. The nutrients in the diluted digests were determined by atomic absorption spectrophotometry using a GBC Avanta spectrophotometer (GBC Scientific Equipment Pty Ltd, Surrey, UK). The results were expressed as mg of EMi (Zn, Fe, Mn, and Cu) per kg dry weight (ppm).

**Figure 3:**
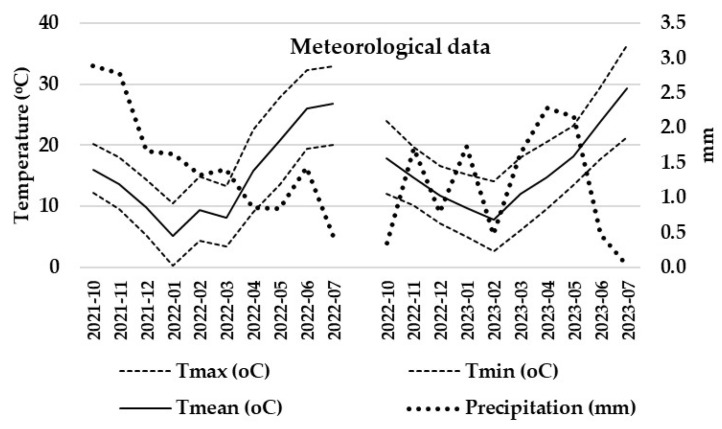
Meteorological data.

The meteorological data are depicted in Figure 3.

The treatments are provided in Table 12. The results of the first year, where SW7 was used as a surfactant, encouraged us to try two more and different surfactants, those of Saldo and Phillon. The interventions included EMis as sulfates, alone or in combination with cysteine (CJ CheilJedang, Seoul, Republic of Korea) or methionine (CJ CheilJedang, Seoul, Republic of Korea). Thus, applications of copper sulfate (0.31 mM), ferrous sulfate (8.93 mM), zinc sulfate (0.31 mM), and manganese sulfate (3.64 mM) were carried out in combination with or without the addition of methionine (5 mM) or cysteine (5 mM). The FytoAmino-Bo (prepared by D. Dimitriadi at Karvelas AVEE, Ypato, Greece) product’s composition was as follows: 2% *w*/*w* Zn sulfate, 5% ethanolamine borate, 1% sodium molybdate, and 5% L-arginine. In the application solution of FABo, zinc sulfate was 0.31 mM. The water used was a drinkable, commercial one (Marata (Sklavenitis); source: “Hitos” AVEE, Kranoula Ioanninon, Greece) with the composition presented in Table 13.

The surfactants used were the following. SW7 (Omex Ltd., Norfolk, Great Britain; distributor: KARVELAS AVEE, Ypato, Greece): It is an ethoxylated organosilicate formulation containing 6.5% *w*/*v* silicon (Si), and the applied dosage was 1 mL of wetting agent per L of application solution (0.1% *v*/*v*). Saldo (Saldo Plus 15 SL, SEGE SA, Athens Greece): It contained ethoxylated isodecyl alcohol 15% *w*/*v*, and the applied dosage was 0.3 mL of wetting agent per L of application solution (0.03% *v*/*v*). Phillon (prepared by D. Dimitriadi at KARVELAS AVEE, Ypato, Greece): This product contains in its composition soybean oil, and the wetting agent incorporated in the formulation was Lutensol T08 (BASF SA, Athens, Greece)—a non-ionic wetting agent and emulsifier made from a saturated iso-C13 alcohol containing 8 moles of ethylene oxide. The applied dosage was 1 mL of product per L of application solution (0.1% *v*/*v*).

### Statistical Analysis

Analysis of variance (ANOVA) was used to explore the effects of various treatments on the trait of interest. Due to multiple testing, Tukey’s “Honest Significant Difference” method [57] was used to control the 95% family-wise confidence level. Significance tests were declared significant when the *p*-value < 0.05. All analyses were performed using the R statistical software (Version 4.0.0) [58].

## 5. Conclusions

Depending on the EMi (Zn, Fe, Mn, or Cu) applied as a sulfate, the studied S-assisted agronomic biofortification strategy provided increases in Zn which were accompanied by (i) a decrease in Cu, (ii) an increase or no increase in Fe, and (iii) a variable change in Mn, with variable intensity. Thus, the increases in Zn concentration were accompanied by reverberations in the concentrations of each of the other three EMis. In molar terms, the EMi metallome per grain was increased by many interventions, and the percentage contribution of each EMi within the metallome was affected by the treatment, whilst the result was ingredient-specific. The reaction of spikes to the interventions presented an acclimation response that included maintenance of the grain weight, but a decrease in the grain weight per spike and yield. Instead, the sum of the accumulated EMis has been increased in several cases, with alteration of the contribution of each EMi to the metallome.

## Figures and Tables

**Table 1:** The effect of interventions containing ZnSO_4_ on grain Zn, Fe, Mn, and Cu concentrations (experimental year 2022–2023). Mean: mean values, SD: standard deviation; SignC: significance code, ns: not statistically significant; * < 0.05; ** < 0.01; *** < 0.001; Δ%: relative percentage difference between the treatment and the control; GS: grading scale—A+ (0 to 19%), B+ (20 to 39%), C+ (40 to 59%), and D+ (>60%), A− (−1 to −19%), B− (−20 to −39%), C− (−40 to −59%), and D− (<−60%).

	Zn (mg kg^−1^)					Fe (mg kg^−1^)				
Intervention	Mean	SD	SignC	Δ%	GS	Mean	SD	SignC	Δ%	GS
Control	27.20	0.78				23.85	0.73			
ZnSO_4_	36.68	0.33	***	35	B+	23.40	1.17	ns	−2	A−
ZnSO_4_/SW7	36.16	0.65	***	33	B+	26.58	1.28	*	11	A+
ZnSO_4_/Saldo	39.82	6.47	*	46	C+	27.31	2.23	ns	15	A+
ZnSO_4_/Phillon	38.97	2.48	**	43	C+	27.92	6.14	ns	17	A+
ZnSO_4_/Cys	40.67	4.38	**	50	C+	29.93	3.41	**	26	B+
ZnSO_4_/Cys/SW7	38.51	1.50	***	42	C+	32.67	2.96	**	37	B+
ZnSO_4_/Cys/Saldo	34.52	0.52	***	27	B+	30.49	1.45	**	28	B+
ZnSO_4_/Cys/Phillon	42.89	5.88	**	58	C+	33.79	5.86	**	42	C+
ZnSO_4_/Met	38.44	4.77	*	41	C+	28.20	2.12	ns	18	A+
ZnSO_4_/Met/SW7	34.59	4.12	*	27	B+	26.58	2.40	ns	11	A+
ZnSO_4_/Met/Saldo	34.59	4.12	*	27	B+	25.80	2.68	ns	8	A+
ZnSO_4_/Met/Phillon	35.89	3.07	*	32	B+	28.82	1.17	**	21	B+
	Mn (mg kg^−1^)					Cu (mg kg^−1^)				
Intervention	mean	SD	SignC	Δ%	GS	mean	SD	SignC	Δ%	GS
Control	9.39	0.22				5.72	0.89			
ZnSO_4_	7.36	4.07	ns	−22	B−	4.00	0.25	*	−30	B−
ZnSO_4_/SW7	9.17	3.85	ns	−2	A−	4.00	0.13	*	−30	B−
ZnSO_4_/Saldo	8.46	4.61	ns	−10	A−	4.07	0.76	*	−29	B−
ZnSO_4_/Phillon	6.81	1.48	*	−27	B−	3.50	0.51	*	−39	B−
ZnSO_4_/Cys	12.03	1.10	*	28	B+	3.75	0.51	*	−34	B−
ZnSO_4_/Cys/SW7	10.00	4.01	ns	6	A+	3.56	0.44	*	−38	B−
ZnSO_4_/Cys/Saldo	10.22	2.58	ns	9	A+	3.69	0.25	*	−36	B−
ZnSO_4_/Cys/Phillon	9.78	2.58	ns	4	A+	3.81	0.06	*	−33	B−
ZnSO_4_/Met	7.31	3.24	ns	−22	B−	4.13	0.38	*	−28	B−
ZnSO_4_/Met/SW7	5.93	1.65	*	−37	B−	3.88	0.64	*	−32	B−
ZnSO_4_/Met/Saldo	8.74	1.43	ns	−7	A−	3.50	0.44	*	−39	B−
ZnSO_4_/Met/Phillon	9.83	1.15	ns	5	A+	3.43	0.25	*	−40	C−

**Table 2:** The effect of interventions containing FeSO_4_ on grain Zn, Fe, Mn, and Cu concentrations (experimental year 2022–2023). Mean: mean values, SD: standard deviation; SignC: significance code, ns: not statistically significant; * < 0.05; ** < 0.01; *** < 0.001; Δ%: relative percentage difference between the treatment and the control; GS: grading scale—A+ (0 to 19%), B+ (20 to 39%), C+ (40 to 59%), and D+ (>60%), A− (−1 to −19%), B− (−20 to −39%), C− (−40 to −59%), and D− (<−60%).

	Zn (mg kg^−1^)					Fe (mg kg^−1^)				
Intervention	Mean	SD	SignC	Δ%	GS	Mean	SD	SignC	Δ%	GS
Control	27.20	0.78				23.85	0.73			
FeSO_4_	34.32	0.59	**	26	B+	23.51	4.02	ns	−1	A−
FeSO_4_/SW7	35.11	1.05	**	29	B+	25.07	2.01	ns	5	A+
FeSO_4_/Saldo	34.06	0.13	**	25	B+	26.69	0.28	ns	12	A+
FeSO_4_/Phillon	39.23	2.75	*	44	C+	29.26	2.62	ns	23	B+
FeSO_4_/Cys	38.12	0.78	***	40	C+	23.90	0.45	ns	0	A+
FeSO_4_/Cys/SW7	31.84	0.13	*	17	A+	24.91	0.56	ns	4	A+
FeSO_4_/Cys/Saldo	30.66	3.27	***	13	A+	25.86	4.13	**	8	A+
FeSO_4_/Cys/Phillon	36.22	1.18	***	33	B+	25.13	2.18	ns	5	A+
FeSO_4_/Met	37.20	0.98	**	37	B+	27.14	1.68	*	14	A+
FeSO_4_/Met/SW7	30.73	1.90	***	13	A+	26.19	4.36	*	10	A+
FeSO_4_/Met/Saldo	36.87	0.98	**	36	B+	23.85	1.34	ns	0	A+
FeSO_4_/Met/Phillon	36.02	1.05	**	32	B+	27.92	2.96	**	17	A+
	Mn (mg kg^−1^)					Cu (mg kg^−1^)				
Intervention	mean	SD	SignC	Δ%	GS	mean	SD	SignC	Δ%	GS
Control	9.39	0.22				5.72	0.89			
FeSO_4_	8.52	2.80	**	−9	A−	3.88	0.38	*	−32	B−
FeSO_4_/SW7	7.97	3.68	*	−15	A−	4.07	0.13	ns	−29	B−
FeSO_4_/Saldo	10.88	0.55	*	16	A+	4.13	0.25	ns	−28	B−
FeSO_4_/Phillon	10.93	3.63	**	16	A+	4.19	0.70	*	−27	B−
FeSO_4_/Cys	9.28	0.33	**	−1	A−	3.62	0.38	*	−37	B−
FeSO_4_/Cys/SW7	10.77	3.90	***	15	A+	3.37	0.32	*	−41	C−
FeSO_4_/Cys/Saldo	7.75	0.38	***	−18	A−	3.56	0.25	*	−38	B−
FeSO_4_/Cys/Phillon	6.81	2.09	***	−27	B−	4.07	0.06	*	−29	B−
FeSO_4_/Met	6.54	0.16	*	−30	B−	4.13	0.38	*	−28	B−
FeSO_4_/Met/SW7	8.95	3.68	**	−5	A−	4.00	0.19	*	−30	B−
FeSO_4_/Met/Saldo	7.86	3.46	***	−16	A−	4.38	0.38	*	−23	B−
FeSO_4_/Met/Phillon	8.68	3.46	***	−8	A−	4.07	0.13	ns	−29	B−

**Table 3:** The effect of interventions containing MnSO_4_ on grain Zn, Fe, Mn, and Cu concentrations (experimental year 2022–2023). Mean: mean values, SD: standard deviation; SignC: significance code, ns: not statistically significant; * < 0.05; ** < 0.01; *** < 0.001; Δ%: relative percentage difference between the treatment and the control; GS: grading scale—A+ (0 to 19%), B+ (20 to 39%), C+ (40 to 59%), and D+ (>60%), A− (−1 to −19%), B− (−20 to −39%), C− (−40 to −59%), and D− (<−60%).

	Zn (mg kg^−1^)					Fe (mg kg^−1^)				
Intervention	Mean	SD	SignC	Δ%	GS	Mean	SD	SignC	Δ%	GS
Control	27.20	0.78				23.85	0.73			
MnSO_4_	41.71	0.92	*	53	C+	30.49	4.36	ns	28	B+
MnSO_4_/SW7	46.62	5.17	**	71	D+	33.00	0.95	**	38	B+
MnSO_4_/Saldo	30.53	0.78	**	12	A+	32.28	3.18	ns	35	B+
MnSO_4_/Phillon	30.92	0.78	**	14	A+	27.92	3.85	ns	17	A+
MnSO_4_/Cys	30.01	1.18	**	10	A+	23.57	1.79	ns	−1	A−
MnSO_4_/Cys/SW7	35.24	0.52	***	30	B+	24.40	0.84	ns	2	A+
MnSO_4_/Cys/Saldo	31.45	1.57	*	16	A+	22.11	0.50	*	−7	A−
MnSO_4_/Cys/Phillon	31.71	1.18	**	17	A+	23.79	0.56	ns	0	A+
MnSO_4_/Met	27.39	1.77	ns	1	A+	24.01	1.06	ns	1	A+
MnSO_4_/Met/SW7	25.76	1.63	ns	−5	A−	23.34	1.62	ns	−2	A−
MnSO_4_/Met/Saldo	28.70	0.65	*	6	A+	22.34	2.40	ns	−6	A−
MnSO_4_/Met/Phillon	30.34	1.11	**	12	A+	25.86	1.17	ns	8	A+
	Mn (mg kg^−1^)					Cu (mg kg^−1^)				
Intervention	mean	SD	SignC	Δ%	GS	mean	SD	SignC	Δ%	GS
Control	9.39	0.22				5.72	0.89			
MnSO_4_	11.48	5.44	ns	22	B+	3.69	0.38	*	−36	B−
MnSO_4_/SW7	14.39	5.05	ns	53	C+	4.00	0.38	*	−30	B−
MnSO_4_/Saldo	13.29	5.16	ns	42	C+	3.37	0.38	*	−41	C−
MnSO_4_/Phillon	12.25	5.33	ns	30	B+	3.81	0.38	*	−33	B−
MnSO_4_/Cys	8.68	1.54	ns	−8	A−	3.50	0.38	*	−39	B−
MnSO_4_/Cys/SW7	10.22	3.74	ns	9	A+	3.75	0.13	*	−34	B−
MnSO_4_/Cys/Saldo	14.17	3.74	*	51	C+	3.37	0.64	*	−41	C−
MnSO_4_/Cys/Phillon	8.68	1.54	ns	−8	A−	3.88	0.44	*	−32	B−
MnSO_4_/Met	8.85	2.58	ns	−6	A−	3.37	0.38	*	−41	C−
MnSO_4_/Met/SW7	15.93	0.49	***	70	B+	3.24	0.06	*	−43	C−
MnSO_4_/Met/Saldo	13.24	4.23	ns	41	C+	3.30	0.57	*	−42	C−
MnSO_4_/Met/Phillon	12.69	2.86	ns	35	B+	3.94	0.06	*	−31	B−

**Table 4:** The effect of interventions containing CuSO_4_ on grain Zn, Fe, Mn, and Cu concentrations (experimental year 2022–2023). Mean: mean values, SD: standard deviation; SignC: significance code, ns: not statistically significant; * < 0.05; ** < 0.01; *** < 0.001; Δ%: relative percentage difference between the treatment and the control; GS: grading scale—A+ (0 to 19%), B+ (20 to 39%), C+ (40 to 59%), and D+ (>60%), A− (−1 to −19%), B− (−20 to −39%), C− (−40 to −59%), and D− (<−60%).

	Zn (mg kg^−1^)					Fe (mg kg^−1^)				
Intervention	Mean	SD	SignC	Δ%	GS	Mean	SD	SignC	Δ%	GS
Control	27.20	0.78				23.85	0.73			
CuSO_4_	37.53	3.73	***	38	B+	35.68	3.02	ns	50	C+
CuSO_4_/SW7	46.94	4.38	***	73	D+	27.48	3.07	ns	15	A+
CuSO_4_/Saldo	45.44	5.30	***	67	D+	28.76	3.24	**	21	B+
CuSO_4_/Phillon	41.97	8.96	**	54	C+	31.38	4.69	*	32	B+
CuSO_4_/Cys	35.31	1.31	***	30	B+	28.43	2.79	ns	19	A+
CuSO_4_/Cys/SW7	35.57	1.44	***	31	B+	27.81	2.74	ns	17	A+
CuSO_4_/Cys/Saldo	40.14	1.77	**	48	C+	29.71	1.90	ns	25	B+
CuSO_4_/Cys/Phillon	39.69	1.90	***	46	C+	36.97	11.73	ns	55	C+
CuSO_4_/Met	43.22	4.25	***	59	C+	28.70	1.90	ns	20	B+
CuSO_4_/Met/SW7	39.75	2.03	*	46	C+	28.15	2.29	ns	18	A+
CuSO_4_/Met/Saldo	35.50	1.83	***	31	B+	31.94	6.70	ns	34	B+
CuSO_4_/Met/Phillon	31.06	0.85	***	14	A+	27.20	0.78	ns	14	A+
	Mn (mg kg^−1^)					Cu (mg kg^−1^)				
Intervention	mean	SD	SignC	Δ%	GS	mean	SD	SignC	Δ%	GS
Control	9.39	0.22				5.72	0.89			
CuSO_4_	6.59	0.82	ns	−30	B−	3.81	0.38	*	−33	B−
CuSO_4_/SW7	7.58	0.77	ns	−19	A−	5.59	0.13	*	−2	A−
CuSO_4_/Saldo	7.80	0.55	*	−17	A−	6.29	0.89	*	10	A+
CuSO_4_/Phillon	7.58	0.27	ns	−19	A−	3.18	0.38	*	−44	C−
CuSO_4_/Cys	7.75	0.27	ns	−18	A−	4.13	0.38	*	−28	B−
CuSO_4_/Cys/SW7	5.38	0.44	ns	−43	C−	4.13	0.13	*	−28	B−
CuSO_4_/Cys/Saldo	6.87	0.22	**	−27	A−	4.19	0.13	*	−27	B−
CuSO_4_/Cys/Phillon	6.54	0.16	ns	−30	B−	3.81	0.06	*	−33	B−
CuSO_4_/Met	8.46	0.33	***	−10	A−	3.81	0.38	*	−33	B−
CuSO_4_/Met/SW7	6.15	0.99	ns	−35	B−	3.69	0.13	*	−36	B−
CuSO_4_/Met/Saldo	7.75	0.22	ns	−18	A−	3.81	0.13	ns	−33	B−
CuSO_4_/Met/Phillon	7.53	0.22	ns	−20	B−	4.38	0.13	*	−23	B−

**Table 5:** The effect of interventions containing FABo on grain Zn, Fe, Mn, and Cu concentrations (experimental year 2022–2023). Mean: mean values, SD: standard deviation; SignC: significance code, ns: not statistically significant; * < 0.05; ** < 0.01; *** < 0.001; Δ%: relative percentage difference between the treatment and the control; GS: grading scale—A+ (0 to 19%), B+ (20 to 39%), C+ (40 to 59%), and D+ (>60%), A− (−1 to −19%), B− (−20 to −39%), C− (−40 to −59%), and D− (<−60%).

	Zn (mg kg^−1^)					Fe (mg kg^−1^)				
Intervention	Mean	SD	SignC	Δ%	GS	Mean	SD	SignC	Δ%	GS
Control	27.20	0.78				23.85	0.73			
FABo	46.94	2.35	**	73	D+	31.50	2.51	**	32	B+
FABo/SW7	35.11	0.92	**	29	B+	30.38	4.08	*	27	B+
FABo/Saldo	42.37	5.10	*	56	C+	29.71	3.24	*	25	B+
FABo/Phillon	33.21	5.49	**	22	B+	30.21	4.58	*	27	B+
FABo/Cys	30.66	0.85	*	13	A+	28.76	0.95	**	21	B+
FABo/Cys/SW7	29.42	0.52	***	8	A+	30.94	1.95	**	30	B+
FABo/Cys/Saldo	37.53	0.98	**	38	B+	32.39	2.51	**	36	B+
FABo/Cys/Phillon	44.52	1.05	**	64	D+	30.04	5.53	*	26	B+
FABo/Met	37.14	1.24	**	37	B+	30.38	0.89	**	27	B+
FABo/Met/SW7	38.44	4.18	*	41	C+	32.11	3.13	**	35	B+
FABo/Met/Saldo	39.03	1.96	**	44	C+	30.88	1.90	**	30	B+
FABo/Met/Phillon	39.82	1.44	*	46	C+	32.61	1.79	**	37	B+
	Mn (mg kg^−1^)					Cu (mg kg^−1^)				
Intervention	mean	SD	SignC	Δ%	GS	mean	SD	SignC	Δ%	GS
Control	9.39	0.22				5.72	0.89			
FABo	11.32	1.65	ns	20	B+	3.69	0.38	ns	−36	B−
FABo/SW7	8.02	0.60	*	−15	A−	3.81	0.13	ns	−33	B−
FABo/Saldo	6.98	0.16	***	−26	B−	3.56	0.13	*	−38	B−
FABo/Phillon	6.92	0.77	***	−26	B−	3.24	0.06	*	−43	C−
FABo/Cys	7.58	0.22	***	−19	A−	1.97	0.38	***	−66	D−
FABo/Cys/SW7	8.95	0.71	ns	−5	A−	2.61	0.19	*	−54	C−
FABo/Cys/Saldo	9.12	0.27	ns	−3	A−	1.59	0.02	***	−72	D−
FABo/Cys/Phillon	6.37	1.21	*	−32	B−	4.26	0.32	ns	−26	B−
FABo/Met	7.58	0.49	***	−19	A−	3.05	0.38	*	−47	C−
FABo/Met/SW7	8.46	0.49	ns	−10	A−	2.86	0.06	*	−50	C−
FABo/Met/Saldo	10.22	0.05	*	9	A+	2.54	0.25	*	−56	C−
FABo/Met/Phillon	8.79	0.77	ns	−6	A−	2.22	0.00	**	−61	D−

**Table 6:** The effect of interventions containing the additives on grain Zn, Fe, Mn, and Cu concentrations (experimental year 2022–2023). Mean: mean values, SD: standard deviation; SignC: significance code, ns: not statistically significant; * < 0.05; ** < 0.01; *** < 0.001; Δ%: relative percentage difference between the treatment and the control; GS: grading scale—A+ (0 to 19%), B+ (20 to 39%), C+ (40 to 59%), and D+ (>60%), A− (−1 to −19%), B− (−20 to −39%), C− (−40 to −59%), and D− (<−60%).

	Zn (mg kg^−1^)					Fe (mg kg^−1^)				
Intervention	Mean	SD	SignC	Δ%	GS	Mean	SD	SignC	Δ%	GS
Control	27.20	0.78				23.85	0.73			
SW7	28.18	1.18	ns	4	A+	21.78	1.95	ns	−9	A−
Saldo	27.66	1.70	ns	2	A+	23.34	1.34	ns	−2	A−
Phillon	27.13	1.44	ns	0	A+	25.97	3.80	ns	9	A+
Cys	42.30	0.92	**	56	C+	29.88	6.48	ns	25	B+
Cys/SW7	33.28	1.24	**	22	B+	26.41	1.62	ns	11	A+
Cys/Saldo	32.04	0.39	***	18	A+	23.79	2.01	ns	0	A+
Cys/Phillon	31.71	0.78	***	17	A+	23.45	1.40	ns	−2	A−
Met	30.53	0.78	**	12	A+	27.31	3.52	ns	15	A+
Met/SW7	32.49	1.44	**	19	A+	24.96	1.17	ns	5	A+
Met/Saldo	31.84	0.59	***	17	A+	25.86	1.90	ns	8	A+
Met/Phillon	36.09	2.81	**	33	B+	29.54	3.57	ns	24	B+
	Mn (mg kg^−1^)					Cu (mg kg^−1^)				
Intervention	mean	SD	SignC	Δ%	GS	mean	SD	SignC	Δ%	GS
Control	9.39	0.22				5.72	0.89			
SW7	8.68	0.55	ns	−8	A−	4.77	0.25	ns	−17	A−
Saldo	8.90	0.60	ns	−5	A−	5.34	0.95	ns	−7	A−
Phillon	9.01	2.53	ns	−4	A−	4.07	0.38	*	−29	B−
Cys	4.83	1.32	**	−49	C−	4.26	0.32	*	−26	B−
Cys/SW7	7.86	0.38	**	−16	A−	4.07	0.25	*	−29	B−
Cys/Saldo	6.70	1.98	ns	−29	B−	3.43	0.06	*	−40	C−
Cys/Phillon	7.97	0.44	ns	−15	A−	3.81	0.13	*	−33	B−
Met	7.75	0.33	**	−18	A−	3.81	0.06	*	−33	B−
Met/SW7	7.91	0.49	**	−16	A−	3.75	0.38	*	−34	B−
Met/Saldo	6.87	2.25	ns	−27	B−	3.56	0.13	*	−38	B−
Met/Phillon	6.48	0.16	***	−31	B−	4.13	0.25	*	−28	B−

**Table 7:** (**a**). Grain weight and grain weight per spike as affected by the interventions that included additives, ZnSO_4_, and FeSO_4_ (experimental year 2022–2023). (**b**) Grain weight and grain weight per spike as affected by the interventions that included MnSO_4_, CuSO_4_, and FABo (experimental year 2022–2023). Mean: mean values, SD: standard deviation; SignC: significance code, ns: not statistically significant; * < 0.05; ** < 0.01; *** < 0.001; Δ%: relative percentage difference between the treatment and the control; GS: grading scale—A+ (0 to 19%), B+ (20 to 39%), C+ (40 to 59%), and D+ (>60%), A− (−1 to −19%), B− (−20 to −39%), C− (−40 to −59%), and D− (<−60%).

(**a**)										
	Grain weight (g)					Grain weight per spike (g)				
Intervention	mean	SD	Δ%	SignC	GS	mean	SD	Δ%	SignC	GS
Control	0.055	0.010				1.968	0.374			
SW7	0.055	0.005	0	ns	A+	1.717	0.520	−13	ns	A−
Saldo	0.055	0.003	0	ns	A+	1.693	0.351	−14	ns	A−
Phillon	0.052	0.004	−5	ns	A−	1.419	0.302	−28	*	B−
Cys	0.056	0.004	2	ns	A+	1.462	0.513	−26	ns	B−
Cys/SW7	0.054	0.005	−2	ns	A−	1.799	0.565	−9	ns	A−
Cys/Saldo	0.051	0.007	−7	ns	A−	1.699	0.443	−14	ns	A−
Cys/Phillon	0.057	0.005	4	ns	A+	1.596	0.451	−19	ns	A−
Met	0.056	0.005	2	ns	A+	1.502	0.356	−24	ns	B−
Met/SW7	0.056	0.005	2	ns	A+	1.864	0.469	−5	ns	A−
Met/Saldo	0.06	0.003	9	ns	A+	1.896	0.621	−4	ns	A−
Met/Phillon	0.054	0.005	−2	ns	A−	1.523	0.726	−23	ns	B−
ZnSO_4_	0.054	0.008	−2	ns	A−	1.713	0.605	−13	ns	A−
ZnSO_4_/SW7	0.056	0.003	2	ns	A+	1.764	0.425	−10	ns	A−
ZnSO_4_/Saldo	0.056	0.004	2	ns	A+	2.109	0.553	7	ns	A+
ZnSO_4_/Phillon	0.056	0.004	2	ns	A+	1.704	0.574	−13	ns	A−
ZnSO_4_/Cys	0.057	0.002	4	ns	A+	1.732	0.438	−12	ns	A−
ZnSO_4_/Cys/SW7	0.055	0.007	0	ns	A+	2.004	0.371	2	ns	A+
ZnSO_4_/Cys/Saldo	0.057	0.005	4	ns	A+	1.971	0.702	0	ns	A+
ZnSO_4_/Cys/Phillon	0.057	0.006	4	ns	A+	1.876	0.239	−5	ns	A−
ZnSO_4_/Met	0.051	0.007	−7	ns	A−	1.74	0.549	−12	ns	A−
ZnSO_4_/Met/SW7	0.057	0.004	4	ns	A+	2.039	0.353	4	ns	A+
ZnSO_4_/Met/Saldo	0.055	0.005	0	ns	A+	1.624	0.495	−17	ns	A−
ZnSO_4_/Met/Phillon	0.055	0.004	0	ns	A+	1.288	0.376	−35	***	B−
FeSO_4_	0.057	0.022	4	ns	A+	1.616	0.643	−18	ns	A−
FeSO_4_/SW7	0.06	0.023	9	ns	A+	1.488	0.571	−24	ns	B−
FeSO_4_/Saldo	0.059	0.023	7	ns	A+	1.566	0.432	−20	ns	B−
FeSO_4_/Phillon	0.055	0.009	0	ns	A+	1.656	0.565	−11	ns	A−
FeSO_4_/Cys	0.069	0.047	25	ns	B+	1.555	0.617	−21	ns	B−
FeSO_4_/Cys/SW7	0.056	0.020	2	ns	A+	1.412	0.493	−28	*	B−
FeSO_4_/Cys/Saldo	0.06	0.029	9	ns	A+	1.258	0.499	−36	***	B−
FeSO_4_/Cys/Phillon	0.066	0.038	20	ns	B+	0.965	0.489	−51	***	C−
FeSO_4_/Met	0.073	0.052	33	ns	B+	1.191	0.554	−39	***	B−
FeSO_4_/Met/SW7	0.061	0.028	11	ns	A+	1.818	0.454	−8	ns	A−
FeSO_4_/Met/Saldo	0.051	0.032	−7	ns	A−	1.404	0.613	−29	*	B−
FeSO_4_/Met/Phillon	0.05	0.024	−9	ns	A−	1.379	0.669	−30	**	B−
(**b**)										
	Grain weight (g)					Grain weight per spike (g)				
	mean	SD	Δ%	SignC	GS	mean	SD	Δ%	SignC	GS
Control	0.055	0.010				1.968	0.374	5	ns	
MnSO_4_	0.057	0.010	4	ns	A+	2.072	0.466	5	ns	A+
MnSO_4_/SW7	0.058	0.026	5	ns	A+	1.688	0.604	−14	ns	A−
MnSO_4_/Saldo	0.058	0.018	5	ns	A+	2.011	0.412	2	ns	A+
MnSO_4_/Phillon	0.056	0.017	2	ns	A+	1.958	0.395	−1	ns	A−
MnSO_4_/Cys	0.063	0.026	15	ns	A+	2.123	0.472	8	ns	A+
MnSO_4_/Cys/SW7	0.057	0.019	4	ns	A+	1.730	0.391	−12	ns	A−
MnSO_4_/Cys/Saldo	0.058	0.026	5	ns	A+	1.826	0.497	−7	ns	A−
MnSO_4_/Cys/Phillon	0.059	0.023	7	ns	A+	1.799	0.381	−9	ns	A−
MnSO_4_/Met	0.055	0.019	0	ns	A+	1.429	0.384	−27	*	B−
MnSO_4_/Met/SW7	0.059	0.029	7	ns	A+	1.619	0.491	−18	ns	A−
MnSO_4_/Met/Saldo	0.058	0.020	5	ns	A+	1.847	0.345	−6	ns	A−
MnSO_4_/Met/Phillon	0.055	0.010	0	ns	A+	2.210	0.367	12	ns	A+
CuSO_4_	0.058	0.013	5	ns	A+	1.817	0.394	−8	ns	A−
CuSO_4_/SW7	0.056	0.024	2	ns	A+	1.266	0.366	−36	***	B−
CuSO_4_/Saldo	0.058	0.017	5	ns	A+	1.496	0.321	−24	ns	B−
CuSO_4_/Phillon	0.051	0.013	−7	ns	A−	1.187	0.364	−40	***	C−
CuSO_4_/Cys	0.055	0.027	0	ns	A+	1.570	0.610	−20	ns	B−
CuSO_4_/Cys/SW7	0.054	0.015	−2	ns	A−	1.478	0.398	−25	ns	B−
CuSO_4_/Cys/Saldo	0.074	0.044	35	ns	B+	0.855	0.464	−57	***	C−
CuSO_4_/Cys/Phillon	0.071	0.048	29	ns	B+	0.816	0.415	−59	***	C−
CuSO_4_/Met	0.070	0.057	27	ns	B+	1.298	0.718	−34	***	B−
CuSO_4_/Met/SW7	0.046	0.021	−16	ns	A−	1.049	0.180	−47	***	C−
CuSO_4_/Met/Saldo	0.063	0.052	15	ns	A+	1.306	0.651	−34	***	B−
CuSO_4_/Met/Phillon	0.070	0.043	27	ns	B+	1.648	0.556	−16	ns	A−
FABo	0.053	0.014	−4	ns	A−	1.618	0.725	−18	ns	A−
FABo/SW7	0.059	0.004	7	ns	A+	1.763	0.645	−10	ns	A−
FABo/Saldo	0.050	0.016	−9	ns	A−	0.805	0.549	−59	***	C−
FABo/Phillon	0.057	0.006	4	ns	A+	1.220	0.389	−38	***	B−
FABo/Cys	0.055	0.006	0	ns	A+	1.101	0.419	−44	***	C−
FABo/Cys/SW7	0.059	0.003	7	ns	A+	1.733	0.304	−12	ns	A−
FABo/Cys/Saldo	0.057	0.003	4	ns	A+	1.737	0.477	−12	ns	A−
FABo/Cys/Phillon	0.059	0.006	7	ns	A+	1.493	0.359	−24	ns	B−
FABo/Met	0.057	0.006	4	ns	A+	1.009	0.366	−49	***	C−
FABo/Met/SW7	0.052	0.004	−5	ns	A−	1.864	0.759	−5	ns	A−
FABo/Met/Saldo	0.054	0.007	−2	ns	A−	1.578	0.590	−20	ns	B−
FABo/Met/Phillon	0.056	0.003	2	ns	A+	0.895	0.576	−55	***	C−

**Table 8:** Calculated grain yield as affected by the interventions (experimental year 2022–2023); Δ%: relative percentage difference between the treatment and the control; GS: grading scale—A+ (0 to 19%), B+ (20 to 39%), C+ (40 to 59%), and D+ (>60%), A− (−1 to −19%), B− (−20 to −39%), C− (−40 to −59%), and D− (<−60%). Interventions marked with bold letters presented a positive GS.

Intervention	Yield				Intervention	Yield		
	g m^−2^	Δ%	GS			g m^−2^	Δ%	GS
Control	379				Control	379		
					MnSO_4_	399	5	A+
SW7	334	−12	A−		MnSO_4_/SW7	325	−14	A−
Saldo	326	−14	A−		MnSO_4_/Saldo	387	2	A+
Phillon	273	−28	B−		MnSO_4_/Phillon	377	−1	A−
Cys	281	−26	B−		MnSO_4_/Cys	408	8	A+
Cys/SW7	346	−9	A−		MnSO_4_/Cys/SW7	333	−12	A−
Cys/Saldo	327	−14	A−		MnSO_4_/Cys/Saldo	351	−7	A−
Cys/Phillon	307	−19	A−		MnSO_4_/Cys/Phillon	346	−9	A−
Met	289	−24	B−		MnSO_4_/Met	275	−27	B−
Met/SW7	359	−5	A−		MnSO_4_/Met/SW7	311	−18	A−
Met/Saldo	365	−4	A−		MnSO_4_/Met/Saldo	355	−6	A−
Met/Phillon	293	−23	B−		MnSO_4_/Met/Phillon	425	12	A+
ZnSO_4_	330	−13	A−		CuSO_4_	350	−8	A−
ZnSO_4_/SW7	339	−10	A−		CuSO_4_/SW7	244	−36	B−
ZnSO_4_/Saldo	406	7	A+		CuSO_4_/Saldo	288	−24	B−
ZnSO_4_/Phillon	328	−13	B−		CuSO_4_/Phillon	228	−40	C−
ZnSO_4_/Cys	333	−12	A−		CuSO_4_/Cys	302	−20	B−
ZnSO_4_/Cys/SW7	386	2	A+		CuSO_4_/Cys/SW7	284	−25	B−
ZnSO_4_/Cys/Saldo	379	0	A+		CuSO_4_/Cys/Saldo	165	−57	C−
ZnSO_4_/Cys/Phillon	361	−5	A−		CuSO_4_/Cys/Phillon	157	−59	C−
ZnSO_4_/Met	335	−12	A−		CuSO_4_/Met	250	−34	B−
ZnSO_4_/Met/SW7	392	4	**A+**		CuSO_4_/Met/SW7	202	−47	C−
ZnSO_4_/Met/Saldo	312	−18	A−		CuSO_4_/Met/Saldo	251	−34	B−
ZnSO_4_/Met/Phillon	248	−35	B−		CuSO_4_/Met/Phillon	317	−16	A−
FeSO_4_	311	−18	A−		FABo	311	−18	A−
FeSO_4_/SW7	286	−24	B−		FABo/SW7	339	−11	A−
FeSO_4_/Saldo	301	−21	B−		FABo/Saldo	155	−59	C−
FeSO_4_/Phillon	319	−16	A−		FABo/Phillon	235	−38	B−
FeSO_4_/Cys	299	−21	B−		FABo/Cys	212	−44	C−
FeSO_4_/Cys/SW7	272	−28	B−		FABo/Cys/SW7	333	−12	A−
FeSO_4_/Cys/Saldo	242	−36	B−		FABo/Cys/Saldo	334	−12	A−
FeSO_4_/Cys/Phillon	186	−51	C−		FABo/Cys/Phillon	287	−24	B−
FeSO_4_/Met	229	−40	C−		FABo/Met	194	−49	C−
FeSO_4_/Met/SW7	350	−8	A−		FABo/Met/SW7	359	−5	A−
FeSO_4_/Met/Saldo	270	−29	B−		FABo/Met/Saldo	304	−20	B−
FeSO_4_/Met/Phillon	265	−30	B−		FABo/Met/Phillon	172	−55	C−

**Table 9:** (**a**). The accumulated amounts of the essential micronutrients (EMis) Zn, Fe, Mn, and Cu per grain for the treatments that included additives, ZnSO_4_, and FeSO_4_ (experimental year 2022–2023). (**b**) The accumulated amounts of Zn, Fe, Mn, and Cu, per grain mass (GW) for the treatments that included MnSO_4_, CuSO_4_, and FABo (experimental year 2022–2023). Δ%: relative percentage difference between the treatment and the control. GS: grading scale—A+ (0 to 19%), B+ (20 to 39%), C+ (40 to 59%), and D+ (>60%), A− (−1 to −19%), B− (−20 to −39%), C− (−40 to −59%), and D− (<−60%).

(**a**)												
	EMi accumulation per grain (in μmol)											
Treatment	Zn			Fe			Mn			Cu		
	μmol	Δ%	GS	μmol	Δ%	GS	μmol	Δ%	GS	μmol	Δ%	GS
Control	0.023			0.023			0.009			0.005		
SW7	0.024	3	A+	0.021	−7	A−	0.009	−3	A−	0.004	−17	A−
Saldo	0.023	1	A+	0.023	0	A+	0.009	−1	A−	0.005	−8	A−
Phillon	0.022	−6	A−	0.024	5	A+	0.009	−5	A−	0.003	−33	B−
Cys	0.039	72	D+	0.033	42	C+	0.005	−40	C−	0.004	−18	A−
Cys/SW7	0.032	39	B+	0.030	30	B+	0.009	0	A+	0.004	−19	A−
Cys/Saldo	0.029	26	B+	0.025	9	A+	0.007	−20	B−	0.003	−36	B−
Cys/Phillon	0.028	20	B+	0.024	4	A+	0.008	−8	A−	0.003	−32	B−
Met	0.026	12	A+	0.027	17	A+	0.008	−14	A−	0.003	−34	B−
Met/SW7	0.030	30	B+	0.027	17	A+	0.009	−4	A−	0.004	−29	B−
Met/Saldo	0.028	23	A+	0.027	17	A+	0.007	−19	A−	0.003	−35	B−
Met/Phillon	0.039	68	D+	0.037	61	D+	0.008	−8	A−	0.005	−9	A−
ZnSO_4_	0.030	32	B+	0.023	−2	A−	0.007	−20	B−	0.003	−32	B−
ZnSO_4_/SW7	0.031	35	B+	0.027	16	A+	0.009	4	A+	0.004	−29	B−
ZnSO_4_/Saldo	0.034	48	C+	0.027	19	A+	0.009	−4	A−	0.004	−28	B−
ZnSO_4_/Phillon	0.033	45	C+	0.028	22	B+	0.007	−23	B−	0.003	−38	B−
ZnSO_4_/Cys	0.034	46	C+	0.029	26	B+	0.012	31	B+	0.003	−36	B−
ZnSO_4_/Cys/SW7	0.033	43	C+	0.033	42	C+	0.010	13	A+	0.003	−37	B−
ZnSO_4_/Cys/Saldo	0.027	17	A+	0.028	21	B+	0.009	5	A+	0.003	−41	C−
ZnSO_4_/Cys/Phillon	0.027	17	A+	0.025	8	A+	0.007	−19	A−	0.002	−51	C−
ZnSO_4_/Met	0.045	97	D+	0.039	69	D+	0.010	14	A+	0.005	0	A+
ZnSO_4_/Met/SW7	0.031	36	B+	0.028	22	B+	0.006	−29	B−	0.004	−28	B−
ZnSO_4_/Met/Saldo	0.034	47	C+	0.030	29	B+	0.010	13	A+	0.004	−29	B−
ZnSO_4_/Met/Phillon	0.048	108	D+	0.045	95	D+	0.016	73	D+	0.005	−6	A−
FeSO_4_	0.030	30	B+	0.024	4	A+	0.009	−2	A−	0.003	−30	B−
FeSO_4_/SW7	0.032	40	C+	0.027	17	A+	0.009	−3	A−	0.004	−23	B−
FeSO_4_/Saldo	0.032	40	C+	0.030	29	B+	0.012	36	B+	0.004	−19	A−
FeSO_4_/Phillon	0.032	41	C+	0.028	23	B+	0.011	19	A+	0.004	−29	B−
FeSO_4_/Cys	0.040	75	D+	0.030	28	B+	0.012	30	B+	0.004	−21	B−
FeSO_4_/Cys/SW7	0.027	19	A+	0.025	9	A+	0.011	22	B+	0.003	−41	C−
FeSO_4_/Cys/Saldo	0.023	−2	A−	0.022	−3	A−	0.007	−25	B−	0.003	−46	C−
FeSO_4_/Cys/Phillon	0.047	102	D+	0.038	64	D+	0.010	16	A+	0.005	8	A+
FeSO_4_/Met	0.042	81	D+	0.035	54	C+	0.009	−3	A−	0.005	−5	B−
FeSO_4_/Met/SW7	0.029	25	B+	0.029	24	B+	0.010	10	A+	0.004	−23	B−
FeSO_4_/Met/Saldo	0.043	86	D+	0.032	41	C+	0.011	21	B+	0.005	5	A+
FeSO_4_/Met/Phillon	0.023	−2	A−	0.020	−11	A−	0.006	−28	B−	0.003	−47	C−
(**b**)												
	EMi accumulation per grain (in μmol)											
Intervention	Zn			Fe			Mn			Cu		
	μmol	Δ%	GS	μmol	Δ%	GS	μmol	Δ%	GS	μmol	Δ%	GS
Control	0.023			0.023			0.009			0.005		
MnSO_4_	0.036	58	C+	0.031	35	B+	0.012	32	B+	0.003	−34	B−
MnSO_4_/SW7	0.041	80	D+	0.034	49	C+	0.015	69	D+	0.004	−27	B−
MnSO_4_/Saldo	0.027	16	A+	0.033	43	C+	0.014	53	C+	0.003	−40	C−
MnSO_4_/Phillon	0.026	15	A+	0.028	22	A+	0.012	39	B+	0.003	−33	B−
MnSO_4_/Cys	0.019	−16	A−	0.018	−23	A−	0.007	−26	B−	0.002	−54	C−
MnSO_4_/Cys/SW7	0.029	24	A+	0.023	1	A+	0.010	10	A+	0.003	−37	B−
MnSO_4_/Cys/Saldo	0.028	23	B+	0.023	2	A+	0.015	69	D+	0.003	−37	B−
MnSO_4_/Cys/Phillon	0.033	46	C+	0.029	28	B+	0.011	21	B+	0.004	−16	A−
MnSO_4_/Met	0.033	42	C+	0.034	46	C+	0.013	40	C+	0.004	−17	A−
MnSO_4_/Met/SW7	0.024	6	A+	0.026	13	A+	0.018	100	D+	0.003	−37	B−
MnSO_4_/Met/Saldo	0.025	11	A+	0.023	1	A+	0.014	55	C+	0.003	−40	C−
MnSO_4_/Met/Phillon	0.021	−9	A−	0.021	−9	A−	0.010	15	A+	0.003	−44	C−
CuSO_4_	0.033	45	C+	0.037	61	D+	0.007	−23	B−	0.003	−30	B−
CuSO_4_/SW7	0.040	75	D+	0.028	20	B+	0.008	−14	A−	0.005	−1	A−
CuSO_4_/Saldo	0.040	75	D+	0.030	30	B+	0.008	−9	A−	0.006	15	A+
CuSO_4_/Phillon	0.044	90	D+	0.038	66	D+	0.009	4	A+	0.003	−32	B−
CuSO_4_/Cys	0.048	107	D+	0.045	95	D+	0.012	38	B+	0.006	14	A+
CuSO_4_/Cys/SW7	0.022	−3	A−	0.020	−11	A−	0.004	−55	C−	0.003	−47	C−
CuSO_4_/Cys/Saldo	0.074	220	D+	0.064	178	D+	0.015	67	D+	0.008	58	C+
CuSO_4_/Cys/Phillon	0.107	367	D+	0.117	409	D+	0.021	134	D+	0.011	112	D+
CuSO_4_/Met	0.050	118	D+	0.039	70	D+	0.012	30	B+	0.005	−9	A−
CuSO_4_/Met/SW7	0.044	93	D+	0.037	60	D+	0.008	−9	A−	0.004	−15	A−
CuSO_4_/Met/Saldo	0.023	−1	A−	0.024	4	A+	0.006	−34	B−	0.003	−50	C−
CuSO_4_/Met/Phillon	0.016	−32	B−	0.016	−30	B−	0.005	−50	B−	0.002	−55	B−
FABo	0.038	65	D+	0.030	30	B+	0.011	21	B+	0.003	−38	B−
FABo/SW7	0.032	38	B+	0.032	40	C+	0.009	−4	A−	0.004	−29	B−
FABo/Saldo	0.038	66	D+	0.031	36	B+	0.007	−17	A−	0.003	−34	B−
FABo/Phillon	0.029	26	B+	0.031	34	B+	0.007	−20	B−	0.003	−42	C−
FABo/Cys	0.023	0	A+	0.025	10	A+	0.007	−25	B−	0.002	−70	D−
FABo/Cys/SW7	0.016	−32	B−	0.019	−16	A−	0.006	−37	B−	0.001	−71	D−
FABo/Cys/Saldo	0.038	65	D+	0.038	66	D+	0.011	22	B+	0.002	−67	D−
FABo/Cys/Phillon	0.046	101	D+	0.037	59	C+	0.008	−12	A−	0.005	−9	A−
FABo/Met	0.055	137	D+	0.052	127	D+	0.013	47	C+	0.005	−8	A−
FABo/Met/SW7	0.061	166	D+	0.060	160	D+	0.016	78	D+	0.005	−6	A−
FABo/Met/Saldo	0.035	51	C+	0.032	39	B+	0.011	20	B+	0.002	−54	C−
FABo/Met/Phillon	0.040	72	D+	0.038	65	D+	0.010	16	A+	0.002	−55	C−

**Table 10:** Synopsis of the effect of the interventions on the grain metallome, in terms of EMi concentrations and accumulations. For each group of interventions, the range of Δ% is provided, thus characterizing the intensity of the effect. The grading scale includes A+ (0 to 19%), B+ (20 to 39%), C+ (40 to 59%), and D+ (>60%), A− (−1 to −19%), B− (−20 to −39%), C− (−40 to −59%), and D− (<−60%).

EMi Concentrations in the Grain (mg kg^−1^)							
Zn (27.20 mg kg^−1^)		Fe (23.85 mg kg^−1^)		Mn (9.39 mg kg^−1^_)_		Cu (5.72 mg kg^−1^_)_	
ZnSO_4_-containing interventions							
B+ (27%)	C+ (50%)	A− (−2%)	C+ (42%)	B− (−37%)	A+ (9%)	C− (−40%)	B− (−28%)
FeSO_4_-containing interventions							
A+ (13%	C+ (40%)	A− (−1%)	B+ (23%)	B− (−30%)	A+ (16%)	C− (−41%)	B− (−23%)
MnSO_4_-containing interventions							
A− (−5%)	D+ (71%)	A− (−7%)	B+ (38%)	A− (−8%)	D+ (70%)	C− (−43%)	B− (−30%)
CuSO_4_-containing interventions							
A+ (14%)	D+ (73%)	A+ (14%)	C+ (55%)	A− (−17%)	C− (−43%)	C− (−44%)	A− (−2%)
FABo-containing interventions							
A+ (8%)	D+ (73%)	B+ (21%)	B+ (37%)	B− (−26%)	B+ (20%)	D− (−72%)	B− (−33%)
Interventions containing additives							
A+ (0%)	C+ (56%)	A− (−9%)	B+ (25%)	C− (−49%)	A− (−4%)	C− (−40%)	A− (−7%)
Grain Parameters							
Grain weight (0.055 g)			A− (−7%)	B+ (33%)			
Grain weight per spike (1.968 g)			C− (−59%)	A+ (12%)			
Yield (379 g m^−2^)			C− (−59%)	A+ (7%)			
EMi Accumulations in the Grain (μmol Per Grain)							
SEMi (0.061 μmol)		B− (−30%)		D+ (322%)			
Zn (0.023 μmol)		Fe (0.023 μmol)		Mn (0.009 μmol)		Cu (0.005 μmol)	
ZnSO_4_-containing interventions							
A+ (17%)	D+ (108%)	A− (−2%)	D+ (95%)	B− (−29%)	D+ (73%)	B− (−38%)	A+ (0%)
FeSO_4_-containing interventions							
A− (−2%)	D+ (102%)	A− (−11%)	D+ (64%)	B− (−28%)	B+ (36%)	C− (−47%)	A+ (8%)
MnSO_4_-containing interventions							
A− (−16%)	D+ (80%)	A− (−23%)	C+ (49%)	B− (−26%)	D+ (100%)	C− (−54%)	A− (−16%)
CuSO_4_-containing interventions							
A− (−3%)	D+ (367%)	A− (−11%)	D+ (409%)	C− (−55%)	D+ (134%)	C− (−50%)	C+ (58%)
FABo-containing interventions							
A+ (0%)	D+ (166%)	A− (−16%)	D+ (160%)	B− (−37%)	D+ (78%)	D− (−71%)	A− (−6%)
Interventions containing additives							
A+ (1%)	D+ (72%)	A+ (0%)	D+ (61%)	C− (−49%)	A+ (0%)	B− (−36%)	A− (−8%)

**Table 11:** Dates of agronomic works.

Dates	Agronomic Works
Experimental year 2021–2022	
22 December 2021	Sowing
7 March 2022	Formation of the experimental plots
6 May 2022	Application of interventions
8 June 2022	Harvest day
Experimental year 2022–2023	
4 January 2023	Sowing
24 April 2023	Formation of the experimental plots
28 June 2023	Harvest day
30 June 2023	Harvest day

**Table 12:** Treatments per experimental year.

Experimental year 2021–2022			
Reference	SW7		
Cys	Cys, SW7		
Met	Met, SW7		
ZnSO_4_	ZnSO_4_, SW7		
ZnSO_4_, Cys	ZnSO_4_, Cys, SW7		
ZnSO_4_, Met	ZnSO_4_, Met, SW7		
FeSO_4_	FeSO_4_, SW7		
FeSO_4_, Cys	FeSO_4_, Cys, SW7		
FeSO_4_, Met	FeSO_4_, Met, SW7		
MnSO_4_	MnSO_4_, SW7		
MnSO_4_, Cys	MnSO_4_, Cys, SW7		
MnSO_4_, Met	MnSO_4_, Met, SW7		
CuSO_4_	CuSO_4_, SW7		
CuSO_4_, Cys	CuSO_4_, Cys, SW7		
CuSO_4_, Met	CuSO_4_, Met, SW7		
FABo	FABo, SW7		
FABo, Cys	FABo, Cys, SW7		
FABo, Met	FABo, Met, SW7		
Experimental year 2022–2023			
Reference	SW7	Saldo	Phillon
Cys	Cys, SW7	Cys, Saldo	Cys, Phillon
Met	Met, SW7	Met, Saldo	Met, Phillon
ZnSO_4_	ZnSO_4_, SW7	ZnSO_4_, Saldo	ZnSO_4_, Phillon
ZnSO_4_, Cys	ZnSO_4_, Cys, SW7	ZnSO_4_, Cys, Saldo	ZnSO_4_, Cys, Phillon
ZnSO_4_, Met	ZnSO_4_, Met, SW7	ZnSO_4_, Met, Saldo	ZnSO_4_, Met, Phillon
FeSO_4_	FeSO_4_, SW7	FeSO_4_, Saldo	FeSO_4_, Phillon
FeSO_4_, Cys	FeSO_4_, Cys, SW7	FeSO_4_, Cys, Saldo	FeSO_4_, Cys, Phillon
FeSO_4_, Met	FeSO_4_, Met, SW7	FeSO_4_, Met, Saldo	FeSO_4_, Met, Phillon
MnSO_4_	MnSO_4_, SW7	MnSO_4_, Saldo	MnSO_4_, Phillon
MnSO_4_, Cys	MnSO_4_, Cys, SW7	MnSO_4_, Cys, Saldo	MnSO_4_, Cys, Phillon
MnSO_4_, Met	MnSO_4_, Met, SW7	MnSO_4_, Met, Saldo	MnSO_4_, Met, Phillon
CuSO_4_	CuSO_4_, SW7	CuSO_4_, Saldo	CuSO_4_, Phillon
CuSO_4_, Cys	CuSO_4_, Cys, SW7	CuSO_4_, Cys, Saldo	CuSO_4_, Cys, Phillon
CuSO_4_, Met	CuSO_4_, Met, SW7	CuSO_4_, Met, Saldo	CuSO_4_, Met, Phillon
FABo	FABo, SW7	FABo, Saldo	FABo, Phillon
FABo, Cys	FABo, Cys, SW7	FABo, Cys, Saldo	FABo, Cys, Phillon
FABo, Met	FABo, Met, SW7	FABo, Met, Saldo	FABo, Met, Phillon

**Table 13:** Spray water characteristics.

pH	7.75	Ca^2+^	79.90 mg L^−1^	Cl^−^	5.65 mg L^−1^
Electric conductivity (20 °C)	359 μS cm^−1^	Mg^2+^	4.34 mg L^−1^	SO_4_^2−^	17.00 mg L^−1^
Hardness (as CaCO_3_)	217.45 mg L^−1^	Na^+^	4.61 mg L^−1^	NO_3_^−^	15.15 mg L^−1^
		K^+^	0.89 mg L^−1^	NO_2_^−^	-
		NH_4_^+^	-	CO_3_^2−^	-

## Data Availability

The original contributions presented in this study are included in the article/Supplementary Materials. Further inquiries can be directed at the corresponding author.

## Supplementary Materials

The following supporting information can be downloaded at https://www.mdpi.com/article/10.3390/plants14243759/s1, Table S1. The effect of interventions containing ZnSO_4_ on grain Zn, Fe, Mn, and Cu concentrations (experimental year 2021–2022). Table S2. The effect of interventions containing FeSO_4_ on grain Zn, Fe, Mn, and Cu concentrations (experimental year 2021–2022). Table S3. The effect of interventions containing MnSO_4_ on grain Zn, Fe, Mn, and Cu concentrations (experimental year 2021–2022). Table S4. The effect of interventions containing CuSO_4_ on grain Zn, Fe, Mn, and Cu concentrations (experimental year 2021–2022). Table S5(a). The effect of interventions containing FABo on grain Zn, Fe, Mn, and Cu concentrations (experimental year 2021–2022). Table S5(b). The effect of interventions containing FABo- (without arginine as additive) on grain Zn, Fe, Mn, and Cu concentrations (experimental year 2021–2022). Table S6. The effect of interventions containing additives on grain Zn, Fe, Mn, and Cu concentrations (experimental year 2021–2022). Table S7. Grain weight and grain weight per spike as affected by the interventions (experimental year 2021–2022). Mean: mean values, SD: standard deviation; SignC: significance code, ns: not statistically significant; * < 0.05; ** < 0.01; *** < 0.001; Δ%: relative percentage difference between the treatment and the control; GS: grading scale—A+ (0 to 19%), B+ (20 to 39%), C+ (40 to 59%), and D+ (>60%), A− (−1 to −19%), B− (−20 to −39%), C− (−40 to −59%), and D− (<−60%). Table S8. Calculated grain yield as affected by the interventions (experimental year 2021–2022).; Δ%: relative percentage difference between the treatment and the control; GS: grading scale—A+ (0 to 19%), B+ (20 to 39%), C+ (40 to 59%), and D+ (>60%), A− (−1 to −19%), B− (−20 to −39%), C− (−40 to −59%), and D− (<−60%). Table S9. The accumulated amounts of Zn, Fe, Mn, and Cu per grain mass (GW; in μmol) for the treatments that included additives, ZnSO_4_, FeSO_4_, MnSO_4_, CuSO_4_, FABo, and FABo- (experimental year 2021–2022). Figure S1. The sum of the EMi metallome (in μmol) per grain, along with the percentage contribution of each EMi to this metallome (experimental year 2021–2022).
