# Supplementary material for: Biofortification of Durum Wheat Grain: Interactions Between Micronutrients as Affected by Potential Biofortification Enhancers and Surfactants
Source: Plants (Basel). 2025 Dec 10;14(24):3759. doi: 10.3390/plants14243759 (PMC12737245; doi:10.3390/plants14243759)
Supplement: Supplementary file 1 [file plants-14-03759-s001.zip › plants-4002227-supplementary.pdf]

Article

Biofortification of durum wheat grain: Interactions between micronutrients as affected by potential biofortification enhancers and surfactants

Despina Dimitriadi[1,2], Georgios P. Stylianidis[1], Ioannis Tsirogiannis[1], Styliani N. Chorianopoulou[1,3], Dimitris L. Bouranis[1,3,*]

1 Plant Physiology and Morphology Laboratory, Crop Science Department, Agricultural University of Athens, Iera Odos 75, 11855 Athens, Greece
2 Karvelas AVEE, 80th km Athinon-Lamias, 32200 Ypato, Greece
3 PlanTerra, Institute for Plant Nutrition and Soil Quality, Agricultural University of Athens, Iera Odos 75, 11855 Athens, Greece
* Correspondence: bouranis@aua.gr

Supplementary material

Table S1. The effect of the interventions containing ZnSO4 on grain Zn, Fe, Mn, and Cu concentrations (experimental year 2021-2022). Mean: mean values, SD: standard deviation; SignC: significance code, ns: not statistically significant; * <0.05; ** < 0.01; *** < 0.001; Δ%: relative percentage difference between the treatment and the control one; GS: grading scale. A+ (0 to 19%), B+ (20 to 39%), C+ (40 to 59%), and D+ (>60%), A- (-1 to -19%), B- (-20 to -39%), C- (-40 to -59%), and D- (<-60%).

	Zn (mg kg^{-1})					Fe (mg kg^{-1})				
	mean	SD	SignC	Δ%	GS	mean	SD	SignC	Δ%	GS
Control	24.5	0.45				21.70	1.25			
ZnSO4	33.22	0.8	***	36	B+	19.87	1.9	ns	-8	A-
ZnSO4/SW7	31.13	0.45	***	27	B+	23.33	0.85	ns	8	A+
ZnSO4/Cys	33.89	1.25	***	38	B+	25.47	2.63	**	17	A+
ZnSO4/Cys/SW7	31.9	0.98	***	30	B+	29.01	2.04	***	34	B+
ZnSO4/Met	29.22	2.55	***	19	A+	24.45	0.88	***	13	A+
ZnSO4/Met/SW7	27.78	3.01	*	13	A+	26.44	1.03	***	22	B+
	Mn (mg kg^{-1})					Cu (mg kg^{-1})				
	mean	SD	SignC	Δ%	GS	mean	SD	SignC	Δ%	GS
Control	8.77	0.78				6.56	1.05			
ZnSO4	6.89	1.33	*	-21	B-	4.88	0.98	*	-26	B-
ZnSO4/SW7	8.01	1.98	ns	-9	A-	5.25	1.44	ns	-20	B-
ZnSO4/Cys	10.56	0.87	**	20	B+	3.98	1.08	**	-39	B-
ZnSO4/Cys/SW7	9.08	2.02	ns	4	A+	4.22	1.76	*	-36	B-
ZnSO4/Met	7.48	1.87	ns	-15	A-	4.87	2.33	ns	-26	B-
ZnSO4/Met/SW7	6.67	2.31	*	-24	B-	4.01	0.87	***	-39	B-

Table S2. The effect of the interventions containing FeSO4 on grain Zn, Fe, Mn, and Cu concentrations (experimental year 2021-2022). Mean: mean values, SD: standard deviation; SignC: significance code, ns: not statistically significant; * <0.05; ** < 0.01; *** < 0.001; Δ%: relative percentage difference between the treatment and the control one; GS: grading scale. A+ (0 to 19%), B+ (20 to 39%), C+ (40 to 59%), and D+ (>60%), A- (-1 to -19%), B- (-20 to -39%), C- (-40 to -59%), and D- (<-60%).

	Zn (mg kg^{-1})					Fe (mg kg^{-1})				
	mean	SD	SignC	Δ%	GS	mean	SD	SignC	Δ%	GS
Control	24.5	0.45				21.70	1.25			
FeSO4	36.43	1.74	***	49	C+	22.21	2.05	ns	2	A+

FeSO4/SW7	33.68	1.88	***	37	B+	23.71	1.55	ns	9	A+
FeSO4/Cys	39.01	0.86	***	59	C+	22.89	1.34	ns	5	A+
FeSO4/Cys/SW7	29.98	1.22	***	22	B+	25.01	0.87	***	15	A+
FeSO4/Met	33.73	2.7	***	38	B+	24.77	1.37	**	14	A+
FeSO4/Met/SW7	31.11	2.2	***	27	B+	26.54	3.02	**	22	B+
	Mn (mg kg-1)					Cu (mg kg-1)				
	mean	SD	SignC	Δ%	GS	mean	SD	SignC	Δ%	GS
Control	8.77	0.78				6.56	1.05			
FeSO4	9.45	1.9	ns	8	A-	4.76	0.94	*	-27	B-
FeSO4/SW7	8.82	2.5	ns	1	A-	5.51	2.38	ns	-16	A-
FeSO4/Cys	10.03	0.8	*	14	A-	6.43	1.66	ns	-2	A-
FeSO4/Cys/SW7	8.79	1.45	ns	0	A+	5.07	0.85	*	-23	B-
FeSO4/Met	7.11	2.61	ns	-19	A-	4.98	1.18	*	-24	B-
FeSO4/Met/SW7	8.23	1.12	ns	-6	A-	5.27	1.74	ns	-20	B-

Table S3. The effect of the interventions containing MnSO4 on grain Zn, Fe, Mn, and Cu concentrations (experimental year 2021-2022). Mean: mean values, SD: standard deviation; SignC: significance code, ns: not statistically significant; * <0.05; ** < 0.01; *** < 0.001; Δ%: relative percentage difference between the treatment and the control one; GS: grading scale. A+ (0 to 19%), B+ (20 to 39%), C+ (40 to 59%), and D+ (>60%), A- (-1 to -19%), B- (-20 to -39%), C- (-40 to -59%), and D- (<-60%).

	Zn (mg kg-1)					Mn (mg kg-1)				
	mean	SD	SignC	Δ%	GS	mean	SD	SignC	Δ%	GS
Control	24.5	0.45				21.70	1.25			
MnSO4	37.5	0.67	***	53	C+	28.99	1.23	***	34	B+
MnSO4/SW7	39.36	1.96	***	61	D+	29.50	1.78	***	36	B+
MnSO4/Cys	27.02	1.55	**	10	A+	23.07	0.96	ns	6	A+
MnSO4/Cys/SW7	32.77	0.82	***	34	B+	22.09	1.34	ns	2	A+
MnSO4/Met	23.39	0.97	*	-5	A-	21.88	0.79	ns	1	A+
MnSO4/Met/SW7	22.19	1.12	***	-9	A-	22.84	1.74	ns	5	A+
	Mn (mg kg-1)					Cu (mg kg-1)				
	mean	SD	SignC	Δ%	GS	mean	SD	SignC	Δ%	GS
Control	8.77	0.78				6.56	1.05			
MnSO4	12.98	1.59	***	48	C+	4.86	0.66	**	-26	B-
MnSO4/SW7	14.44	1.88	***	65	D+	4.62	1.32	*	-30	B-
MnSO4/Cys	10.17	3.22	ns	16	A+	4.77	1.45	*	-27	B-
MnSO4/Cys/SW7	12.56	1.63	***	43	C+	4.35	0.98	**	-34	B-
MnSO4/Met	11.78	0.78	***	34	B+	4.01	1.04	**	-39	B-
MnSO4/Met/SW7	16.43	2.92	***	87	D+	3.81	1.25	**	-42	C-

Table S4. The effect of the interventions containing CuSO4 on grain Zn, Fe, Mn, and Cu concentrations (experimental year 2021-2022). Mean: mean values, SD: standard deviation; SignC: significance code, ns: not statistically significant; * <0.05; ** < 0.01; *** < 0.001; Δ%: relative percentage difference between the treatment and the control one; GS: grading scale. A+ (0 to 19%), B+ (20 to 39%), C+ (40 to 59%), and D+ (>60%), A- (-1 to -19%), B- (-20 to -39%), C- (-40 to -59%), and D- (<-60%).

	Zn (mg kg-1)					Fe (mg kg-1)				
	mean	SD	SignC	Δ%	GS	mean	SD	SignC	Δ%	GS
Control	24.5	0.45				21.70	1.25			
CuSO4	23.98	1.15	ns	-2	A-	19.63	1.66	*	-10	
CuSO4/SW7	34.19	2.73	***	40	C+	29.46	2.15	***	36	B+
CuSO4/Cys	42.74	3.62	***	74	D+	23.26	0.78	*	7	A+
CuSO4/Cys/SW7	33.09	1.21	***	35	B+	25.21	2.51	*	16	A+
CuSO4/Met	32.45	1.55	***	32	B+	23.59	0.88	*	9	A+
CuSO4/Met/SW7	38.55	2.25	***	57	C+	23.39	1.36	*	8	A+
	Mn (mg kg-1)					Cu (mg kg-1)				
	mean	SD	SignC	Δ%	GS	mean	SD	SignC	Δ%	GS

Control	8.77	0.78				6.56	1.05			
CuSO4	5.56	1.25	***	-37	B-	3.01	0.27	***	-54	C-
CuSO4/SW7	8.47	1.04	ns	-3	A-	4.89	1.22	*	-25	B-
CuSO4/Cys	6.67	0.79	***	-24	B-	4.47	0.87	**	-32	B-
CuSO4/Cys/SW7	7.52	1.12	*	-14	A-	5.52	0.75	ns	-16	A-
CuSO4/Met	7.38	0.88	*	-16	A-	3.02	0.98	***	-54	C-
CuSO4/Met/SW7	7.01	1.22	*	-20	B-	4.33	1.12	**	-34	B-

Table S5a. The effect of the interventions containing FABo on grain Zn, Fe, Mn, and Cu concentrations (experimental year 2021-2022). Mean: mean values, SD: standard deviation; SignC: significance code, ns: not statistically significant; * <0.05; ** < 0.01; *** < 0.001; Δ%: relative percentage difference between the treatment and the control one; GS: grading scale. A+ (0 to 19%), B+ (20 to 39%), C+ (40 to 59%), and D+ (>60%), A- (-1 to -19%), B- (-20 to -39%), C- (-40 to -59%), and D- (<-60%).

	Zn (mg kg^{-1})					Fe (mg kg^{-1})				
	mean	SD	SignC	Δ%	GS	mean	SD	SignC	Δ%	GS
Control	24.5	0.45				21.70	1.25			
FABo	41.83	1.65	***	71	D+	29.45	0.85	***	36	B+
FABo /SW7	37.12	1.2	***	52	C+	28.32	0.81	***	31	B+
FABo /Cys	31.76	0.78	***	30	B+	31.77	0.96	***	46	C+
FABo /Cys/SW7	28.25	1.34	***	15	A+	29.76	1.45	***	37	B+
FABo /Met	32.09	1.47	***	31	B+	31.22	1.74	***	44	C+
FABo /Met/SW7	31.66	0.69	***	29	B+	30.05	1.04	***	38	B+
	Mn (mg kg^{-1})					Cu (mg kg^{-1})				
	mean	SD	SignC	Δ%	GS	mean	SD	SignC	Δ%	GS
Control	8.77	0.78				6.56	1.05			
FABo	10.44	1.03	*	19	A+	4.57	0.77	**	-30	B-
FABo /SW7	9.37	0.78	ns	7	A+	4.29	0.97	**	-35	B-
FABo /Cys	9.03	1.65	ns	3	A+	3.22	1.66	**	-51	C-
FABo /Cys/SW7	8.88	0.63	ns	1	A+	4.83	1.73	ns	-26	B-
FABo /Met	8.23	1.33	ns	-6	A-	5.21	0.84	*	-21	B-
FABo /Met/SW7	8.97	1.45	ns	2	A+	4.02	1.03	**	-39	B-

Table S5b. The effect of the interventions containing FABo- (without arginine as additive) on grain Zn, Fe, Mn, and Cu concentrations (experimental year 2021-2022). Mean: mean values, SD: standard deviation; SignC: significance code, ns: not statistically significant; * <0.05; ** < 0.01; *** < 0.001; Δ%: relative percentage difference between the treatment and the control one; GS: grading scale. A+ (0 to 19%), B+ (20 to 39%), C+ (40 to 59%), and D+ (>60%), A- (-1 to -19%), B- (-20 to -39%), C- (-40 to -59%), and D- (<-60%).

	Zn (mg kg^{-1})					Fe (mg kg^{-1})				
	mean	SD	SignC	Δ%	GS	mean	SD	SignC	Δ%	GS
Control	24.5	0.45				21.70	1.25			
FABo-	40.23	0.99	***	64	D+	25.56	0.88	***	18	A+
FABo- /SW7	38.88	1.45	***	59	C+	26.85	0.89	***	24	B+
FABo- /Cys	33.01	1.85	***	35	B+	27.11	2.01	***	25	B+
FABo- /Cys/SW7	27.77	2.05	***	13	A+	27.19	1.77	***	25	B+
FABo- /Met	29.45	0.88	***	20	B+	32.03	2.22	***	48	C+
FABo- /Met/SW7	29.87	1.45	***	22	B+	30.77	0.79	***	42	C+
	Mn (mg kg^{-1})					Cu (mg kg^{-1})				
	mean	SD	SignC	Δ%	GS	mean	SD	SignC	Δ%	GS
Control	8.77	0.78				6.56	1.05			
FABo-	9.95	0.99	*	13	A+	5.54	1.66	ns	-16	A-
FABo- /SW7	8.44	0.85	ns	-4	A-	5.57	1.29	ns	-15	A-
FABo- /Cys	8.92	1.22	ns	2	A+	4.47	0.88	**	-32	B-

FABo- /Cys/SW7	7.35	1.98	ns	-16	A-	5.55	0.78	ns	-15	A-
FABo- /Met	8.57	1.78	ns	-2	A-	5.02	1.01	*	-23	B-
FABo- /Met/SW7	8.01	2.13	ns	-9	A-	4.93	1.98	ns	-25	B-

Table S6. The effect of the interventions containing the additives on grain Zn, Fe, Mn, and Cu concentrations (experimental year 2021-2022). Mean: mean values, SD: standard deviation; SignC: significance code, ns: not statistically significant; * <0.05; ** < 0.01; *** < 0.001; Δ%: relative percentage difference between the treatment and the control one; GS: grading scale. A+ (0 to 19%), B+ (20 to 39%), C+ (40 to 59%), and D+ (>60%), A- (-1 to -19%), B- (-20 to -39%), C- (-40 to -59%), and D- (<-60%).

	Zn (mg kg^{-1})					Fe (mg kg^{-1})				
	mean	SD	SignC	Δ%	GS	mean	SD	SignC	Δ%	GS
Control	24.5	0.45				21.70	1.25			
SW7	25.88	1.65	ns	6	A+	20.8	0.33	ns	-4	A-
Cys	35.21	1.88	***	44	C+	25.65	0.87	***	18	A+
Cys/SW7	29.55	0.95	***	21	B+	23.78	1.76	*	10	A+
Met	26.99	1.24	**	10	A+	25.81	1.87	**	19	A+
Met/SW7	27.43	1.78	**	12	A+	23.01	1.25	ns	6	A+
	Mn (mg kg^{-1})					Cu (mg kg^{-1})				
	mean	SD	SignC	Δ%	GS	mean	SD	SignC	Δ%	GS
Control	8.77	0.78				6.56	1.05			
SW7	7.51	1.77	ns	-14	A-	4.78	1.47	*	-27	B-
Cys	5.62	1.32	***	-36	B-	4.21	0.84	**	-36	B-
Cys/SW7	6.56	1.06	**	-25	B-	5.22	1.93	ns	-20	B-
Met	6.88	0.86	**	-22	B-	4.07	1.85	**	-38	B-
Met/SW7	7.14	2.04	ns	-19	A-	4.47	2.21	ns	-32	B-

Table S7. Grain weight and grain weight per spike as affected by the internentions (experimental year 2021-2022). Mean: mean values, SD: standard deviation; SignC: significance code, ns: not statistically significant; * <0.05; ** < 0.01; *** < 0.001; Δ%: relative percentage difference between the treatment and the control one; GS: grading scale: A+ (0 to 19%), B+ (20 to 39%), C+ (40 to 59%), and D+ (>60%), A- (-1 to -19%), B- (-20 to -39%), C- (-40 to -59%), and D- (<-60%).

	Grain weight (g)					Grain weight per spike (g)				
	mean	SD	Δ%	SignC	GS	mean	SD	Δ%	SignC	GS
Control	0.054	0.013				1.745	0.404			
SW7	0.067	0.021	24	ns	B+	1.792	0.123	3	ns	A+
Cys	0.062	0.013	15	ns	A+	1.563	0.163	-10	ns	A-
Cys/SW7	0.055	0.011	2	ns	A_	1.645	0.282	-6	ns	A-
Met	0.064	0.016	19	ns	A+	1.495	0.364	-14	ns	A-
Met/SW7	0.052	0.011	-4	ns	A-	1.718	0.320	-2	ns	A-
ZnSO4	0.056	0.012	4	ns	A+	1.472	0.165	-16	ns	A-
ZnSO4/SW7	0.054	0.022	0	ns	A+	1.364	0.431	-22	ns	B-
ZnSO4/Cys	0.048	0.019	-11	ns	A-	1.331	0.327	-24	*	B-
ZnSO4/Cys/SW7	0.046	0.007	-15	ns	A-	1.773	0.265	2	ns	A+
ZnSO4/Met	0.041	0.015	-24	ns	B-	1.245	0.408	-29	***	B-
ZnSO4/Met/SW7	0.065	0.023	20	ns	B+	1.962	0.502	12	ns	A+
FeSO4	0.056	0.015	4	ns	A+	1.400	0.337	-20	ns	B-

FeSO4/SW7	0.054	0.016	0	ns	A+	1.224	0.269	-30	***	B-
FeSO4/Cys	0.047	0.013	-13	ns	A-	1.356	0.339	-22	ns	B-
FeSO4/Cys/SW7	0.039	0.017	-28	ns	A-	1.180	0.415	-32	***	B-
FeSO4/Met	0.064	0.021	19	ns	A+	1.503	0.317	-14	ns	A-
FeSO4/Met/SW7	0.055	0.012	2	ns	A+	1.882	0.442	8	ns	A+
MnSO4	0.044	0.018	-19	ns	A-	1.608	0.589	-8	ns	A-
MnSO4/SW7	0.042	0.018	-22	ns	A-	1.371	0.501	-21	ns	B-
MnSO4/Cys	0.058	0.017	7	ns	A+	1.786	0.385	2	ns	A+
MnSO4/Cys/SW7	0.052	0.022	-4	ns	A-	1.262	0.412	-28	**	B-
MnSO4/Met	0.049	0.013	-9	ns	A-	1.472	0.350	-16	ns	A-
MnSO4/Met/SW7	0.047	0.013	-13	ns	A-	1.424	0.279	-18	ns	A-
CuSO4	0.041	0.013	-24	ns	A-	1.341	0.382	-23	*	B-
CuSO4/SW7	0.044	0.019	-19	ns	A-	1.136	0.501	-35	***	B-
CuSO4/Cys	0.056	0.015	4	ns	A+	1.273	0.302	-27	**	B-
CuSO4/Cys/SW7	0.046	0.018	-15	ns	A-	1.428	0.515	-18	ns	A-
CuSO4/Met	0.047	0.012	-13	ns	A-	1.216	0.280	-30	***	B-
CuSO4/Met/SW7	0.061	0.022	13	ns	A+	1.378	0.398	-21	ns	B-
FABo	0.045	0.019	-17	ns	A-	1.440	0.584	-17	ns	A-
FABo/SW7	0.044	0.017	-19	ns	A-	1.410	0.597	-19	ns	A-
FABo/Cys	0.045	0.013	-17	ns	A-	1.174	0.330	-33	***	B-
FABo/Cys/SW7	0.058	0.026	7	ns	A+	1.325	0.511	-24	*	B-
FABo/Met	0.038	0.017	-30	ns	B-	0.908	0.303	-48	***	C-
FABo/Met/SW7	0.039	0.014	-28	ns	B-	1.257	0.452	-28	***	B-

Table S8. Calculated grain yield as affected by the interventions (experimental year 2021-2022).; Δ%: relative percentage difference between the treatment and the control one; GS: grading scale: A+ (0 to 19%), B+ (20 to 39%), C+ (40 to 59%), and D+ (>60%), A- (-1 to -19%), B- (-20 to -39%), C- (-40 to -59%), and D- (<-60%).

Intervention	Yield			Intervention	Yield		
	$g\ m^{-2}$	Δ%	GS		$g\ m^{-2}$	Δ%	GS
Control	314			Control	314		
				MnSO4	247	-21	B-
SW7	323	3	A+	MnSO4/SW7	321	2	A+
Cys	281	-10	A-	MnSO4/Cys	227	-28	B-
Cys/SW7	296	-6	A-	MnSO4/Cys/SW7	265	-16	A-
Met	269	-14	A-	MnSO4/Met	256	-18	A-
Met/SW7	309	-2	A-	MnSO4/Met/SW7	241	-23	B-
ZnSO4	265	-16	A-	CuSO4	204	-35	B-
ZnSO4/SW7	246	-22	B-	CuSO4/SW7	229	-27	B-
ZnSO4/Cys	240	-24	B-	CuSO4/Cys	257	-18	A-
ZnSO4/Cys/SW7	319	2	A+	CuSO4/Cys/SW7	219	-30	B-
ZnSO4/Met	224	-29	B-	CuSO4/Met	248	-21	B-
ZnSO4/Met/SW7	353	12	A+	CuSO4/Met/SW7	259	-17	A-
FeSO4	252	-20	B-	FABo	254	-19	A-
FeSO4/SW7	220	-30	B-	FABo/SW7	211	-33	B-
FeSO4/Cys	244	-22	B-	FABo/Cys	239	-24	B-

FeSO4/Cys/SW7	212	-32	B-	FABo/Cys/SW7	163	-48	C-
FeSO4/Met	271	-14	A-	FABo/Met	226	-28	B-
FeSO4/Met/SW7	339	8	A+	FABo/Met/SW7	247	-21	B-

Table S9. The accumulated amounts of Zn, Fe, Mn, and Cu per grain mass (GW; in μmol), for the treatments that include additives, ZnSO4, FeSO4, MnSO4, CuSO4, FABo, and FABo- (experimental year 2021-2022). Δ%: relative percentage difference between the treatment and the control one.

	**GEMi (μmol per grain)**							
	Zn		Fe		Mn		Cu	
	μmol	Δ%	μmol	Δ%	μmol	Δ%	μmol	Δ%
Control	0.020		0.021		0.009		0.006	
SW7	0.027	31	0.025	19	0.009	6	0.005	-10
Cys	0.033	65	0.028	36	0.006	-26	0.004	-26
Cys/SW7	0.025	23	0.023	12	0.007	-24	0.005	-19
Met	0.026	31	0.030	41	0.008	-7	0.004	-26
Met/SW7	0.022	8	0.021	2	0.007	-22	0.004	-34
ZnSO4	0.028	41	0.020	-5	0.007	-19	0.004	-23
ZnSO4/SW7	0.026	27	0.023	8	0.008	-9	0.004	-20
ZnSO4/Cys	0.025	23	0.022	4	0.009	7	0.003	-46
ZnSO4/Cys/SW7	0.022	11	0.024	14	0.008	-12	0.003	-45
ZnSO4/Met	0.018	-9	0.018	-14	0.006	-35	0.003	-44
ZnSO4/Met/SW7	0.028	36	0.031	47	0.008	-8	0.004	-26
FeSO4	0.031	54	0.022	6	0.010	12	0.004	-25
FeSO4/SW7	0.028	37	0.023	9	0.009	1	0.005	-16
FeSO4/Cys	0.028	39	0.019	-8	0.009	0	0.005	-15
FeSO4/Cys/SW7	0.018	-12	0.017	-17	0.006	-28	0.003	-44
FeSO4/Met	0.033	63	0.028	35	0.008	-4	0.005	-10
FeSO4/Met/SW7	0.026	29	0.026	25	0.008	-4	0.005	-18
MnSO4	0.025	25	0.023	9	0.010	21	0.003	-40
MnSO4/SW7	0.025	25	0.022	6	0.011	28	0.003	-45
MnSO4/Cys	0.024	18	0.024	14	0.011	25	0.004	-22
MnSO4/Cys/SW7	0.026	29	0.021	-2	0.012	38	0.004	-36
MnSO4/Met	0.018	-13	0.019	-9	0.011	22	0.003	-45
MnSO4/Met/SW7	0.016	-21	0.019	-8	0.014	63	0.003	-49
CuSO4	0.015	-26	0.014	-31	0.004	-52	0.002	-65
CuSO4/SW7	0.023	14	0.023	11	0.007	-21	0.003	-39
CuSO4/Cys	0.037	81	0.023	11	0.007	-21	0.004	-29
CuSO4/Cys/SW7	0.023	15	0.021	-1	0.006	-27	0.004	-28
CuSO4/Met	0.023	15	0.020	-5	0.006	-27	0.002	-60
CuSO4/Met/SW7	0.036	78	0.026	22	0.008	-10	0.004	-25
FABo	0.029	42	0.024	13	0.009	-1	0.003	-42
FABo/SW7	0.025	23	0.022	6	0.008	-13	0.003	-47
FABo/Cys	0.022	8	0.026	22	0.007	-14	0.002	-59
FABo/Cys/SW7	0.025	24	0.031	47	0.009	9	0.004	-21
FABo/Met	0.019	-8	0.021	1	0.006	-34	0.003	-44
FABo/Met/SW7	0.019	-7	0.021	0	0.006	-26	0.002	-56
FABo-	0.033	61	0.024	16	0.010	11	0.005	-17
FABo-/SW7	0.034	68	0.027	31	0.009	2	0.005	-10
FABo-/Cys	0.026	27	0.025	18	0.008	-4	0.004	-36
FABo-/Cys/SW7	0.021	3	0.024	14	0.007	-24	0.004	-23
FABo-/Met	0.020	0	0.026	23	0.007	-19	0.004	-36
FABo-/Met/SW7	0.025	24	0.030	44	0.008	-7	0.004	-23

Treatment	SEMi	Δ%	Zn%	Fe%	Mn%	Cu%
Control	0.055		37	38	16	10
SW7	0.066	19	40	38	14	8
Cys	0.072	31	46	39	9	6
Cys/SW7	0.059	7	42	39	11	8
Met	0.068	23	39	43	12	6
Met/SW7	0.054	-3	41	40	13	7
ZnSO4	0.060	8	48	33	12	7
ZnSO4/SW7	0.061	9	42	37	13	7
ZnSO4/Cys	0.059	6	42	37	16	5
ZnSO4/Cys/SW7	0.057	3	39	42	13	5
ZnSO4/Met	0.045	-19	41	40	12	7
ZnSO4/Met/SW7	0.070	27	39	44	11	6
FeSO4	0.067	21	46	33	14	6
FeSO4/SW7	0.064	16	43	36	14	7
FeSO4/Cys	0.061	9	46	32	14	8
FeSO4/Cys/SW7	0.045	-19	40	39	14	7
FeSO4/Met	0.075	35	44	38	11	7
FeSO4/Met/SW7	0.065	17	40	40	13	7

Treatment	SEMi	Δ%	Zn%	Fe%	Mn%	Cu%
Control	0.055		37	38	16	10
MnSO4	0.062	12	41	37	17	5
MnSO4/SW7	0.062	11	41	36	18	5
MnSO4/Cys	0.063	14	38	38	17	7
MnSO4/Cys/SW7	0.062	12	42	33	19	6
MnSO4/Met	0.050	-9	35	38	21	6
MnSO4/Met/SW7	0.052	-6	31	37	27	5
CuSO4	0.036	-36	42	41	12	5
CuSO4/SW7	0.056	2	41	41	12	6
CuSO4/Cys	0.071	28	52	33	10	6
CuSO4/Cys/SW7	0.054	-2	43	38	12	7
CuSO4/Met	0.052	-7	45	38	12	4
CuSO4/Met/SW7	0.073	33	49	35	11	6
FABo	0.064	16	45	37	13	5
FABo/SW7	0.058	4	43	39	13	5
FABo/Cys	0.057	3	38	45	13	4
FABo/Cys/SW7	0.070	26	36	44	13	6
FABo/Met	0.049	-12	38	44	12	6
FABo/Met/SW7	0.049	-12	39	43	13	5
FABo-	0.071	28	46	34	14	6
FABo-/SW7	0.075	35	45	37	12	7
FABo-/Cys	0.062	13	41	40	13	6
FABo-/Cys/SW7	0.056	0	37	43	12	8
FABo-/Met	0.057	2	36	46	12	6
FABo-/Met/SW7	0.068	22	37	45	12	6

	60%	-	
	40%	-	59%
	20%	-	39%
	0%	-	19%
	-1%	-	-19%
	-20%	-	-39%

Figure S1. The sum of EMi metalome (in μmol) per grain, along with the percentage contribution of each EMi to this metalome (experimental year 2021-2022).

Supplementary material

Table S1. The effect of the interventions containing ZnSO4 on grain Zn, Fe, Mn, and Cu concentrations (experimental year 2021-2022). **Table S2**. The effect of the interventions containing FeSO4 on grain Zn, Fe, Mn, and Cu concentrations (experimental year 2021-2022). **Table S3**. The effect of the interventions containing MnSO4 on grain Zn, Fe, Mn, and Cu concentrations (experimental year 2021-2022). **Table S4**. The effect of the interventions containing CuSO4 on grain Zn, Fe, Mn, and Cu concentrations (experimental year 2021-2022). **Table S5a**. The effect of the interventions containing FABo on grain Zn, Fe, Mn, and Cu concentrations (experimental year 2021-2022). **Table S5b**. The effect of the interventions containing FABo- (without arginine as additive) on grain Zn, Fe, Mn, and Cu concentrations (experimental year 2021-2022). **Table S6**. The effect of the interventions containing the additives on grain Zn, Fe, Mn, and Cu concentrations (experimental year 2021-2022). **Table S7**. Grain weight and grain weight per spike as affected by the internentions (experimental year 2021-2022). Mean: mean values, SD: standard deviation; SignC: significance code, ns: not statistically significant; * <0.05; ** < 0.01; *** < 0.001; Δ%: relative percentage difference between the treatment and the control one; GS: grading scale: A+ (0 to 19%), B+ (20 to 39%), C+ (40 to 59%), and D+ (>60%), A- (-1 to -19%), B- (-20 to -39%), C- (-40 to -59%), and D- (<-60%). **Table S8**. Calculated grain yield as affected by the interventions (experimental year 2021-2022).; Δ%: relative percentage difference between the treatment and the control one; GS: grading scale: A+ (0 to 19%), B+ (20 to 39%), C+ (40 to 59%), and D+ (>60%), A- (-1 to -19%), B- (-20 to -39%), C- (-40 to -59%), and D- (<-60%). **Table S9**. The accumulated amounts of Zn, Fe, Mn, and Cu per grain mass (GW; in μmol), for the treatments that include additives, ZnSO4, FeSO4, MnSO4, CuSO4, FABo, and FABo- (experimental year 2021-2022).

Figure S1. The sum of EMi metalome (in μmol) per grain, along with the percentage contribution of each EMi to this metalome (experimental year 2021-2022).
